# HtrA2/Omi mitigates NAFLD in high-fat-fed mice by ameliorating mitochondrial dysfunction and restoring autophagic flux

**DOI:** 10.1038/s41420-022-01022-4

**Published:** 2022-04-21

**Authors:** Wei Zhou, Xueting Deng, Xiaolei Zhu, Qinhui Yan, Nan Zhou, Susu Du, Xiaonan Li

**Affiliations:** 1 Department of Child Health Care, Children’s Hospital of Nanjing Medical University, 72 Guangzhou Road, 210008 Nanjing, China; 2 Medical Center for Digestive Diseases, Second Affiliated Hospital of Nanjing Medical University, 121 Jiangjiayuan Road, 210011 Nanjing, China; 3 Institute of Pediatric Research, Nanjing Medical University, Nanjing, China

**Keywords:** Non-alcoholic fatty liver disease, Medical research

## Abstract

Nonalcoholic fatty liver disease (NAFLD) is the most common chronic liver metabolic syndrome which affects millions of people worldwide. Recently, improving mitochondrial function and autophagic ability have been proposed as a means to prevent NAFLD. It has been previously described that high-temperature requirement protein A2 (HtrA2/Omi) favors mitochondrial homeostasis and autophagy in hepatocytes. Thus, we explored the effects of HtrA2/Omi on regulating mitochondrial function and autophagy during NAFLD development. High-fat diet (HFD)-induced NAFLD in mice and free fatty acids (FFAs)-induced hepatocytes steatosis in vitro were established. Adeno-associated viruses (AAV) in vivo and plasmid in vitro were used to restore HtrA2/Omi expression. In this study, we reported that HtrA2/Omi expression considerably decreased in liver tissues from the HFD-induced NAFLD model and in L02 cells with FFA-treated. However, restoring HtrA2/Omi ameliorated hepatic steatosis, confirming by improved serum lipid profiles, glucose homeostasis, insulin resistance, histopathological lipid accumulation, and the gene expression related to lipid metabolism. Moreover, HtrA2/Omi also attenuated HFD-mediated mitochondrial dysfunction and autophagic blockage. TEM analysis revealed that liver mitochondrial structure and autophagosome formation were improved in hepatic HtrA2/Omi administration mice compared to HFD mice. And hepatic HtrA2/Omi overexpression enhanced mitochondrial fatty acid β-oxidation gene expression, elevated LC3II protein levels, induced LC3 puncta, and decreased SQSTM1/p62 protein levels. Furthermore, hepatic HtrA2/Omi increased respiratory exchange ratio and heat production in mice. Finally, HtrA2/Omi overexpression by plasmid significantly diminished lipid accumulation, mitochondrial dysfunction, and autophagic inhibition in FFA-treated L02 hepatocytes. Taken together, we demonstrated that HtrA2/Omi was a potential candidate for the treatment of NAFLD via improving mitochondrial functions, as well as restoring autophagic flux.

## Introduction

Nonalcoholic fatty liver disease (NAFLD) is a common and complex chronic liver disease worldwide, which affects one-fourth of adults globally [1]. NAFLD is characterized by lipid accumulation in hepatocytes and comprises a continuum of liver diseases, ranging from simple steatosis, steatohepatitis, fibrosis, cirrhosis, and liver cancer [2]. In addition, most patients with NAFLD have other metabolic diseases, including cardiovascular diseases and type 2 diabetes mellitus [3]. Accumulated evidence has implicated that the initiation and progression of NAFLD are associated with hepatic lipid deposition, oxidative stress, mitochondrial dysfunction, and endoplasmic reticulum stress [3]. Notably, mitochondrial dysfunction plays a key role in the pathophysiology of NAFLD [4].

Hepatic mitochondria orchestrate energy metabolism homeostasis by tricarboxylic acid cycle, adenosine triphosphate synthesis, and reactive oxygen species (ROS) formation. However, during the progression of NAFLD, the consequential overaccumulation of lipid in hepatocytes results in oxidative stress and lipotoxicity, contributing to mitochondrial oxidative damage and subsequent mitochondrial impairment [5]. Other reports have supported the view that dysfunction mitochondria produce less ATP and more ROS, destroy the fatty acid β-oxidation, release toxic lipid intermediates, and then cause further liver injury [6]. Moreover, dysfunctional mitochondria are accumulated in the livers of patients and mice with NAFLD [7, 8]. Besides, impaired mitochondria promote the assembly of the inflammasome, which exacerbates hepatic damage [9]. Furthermore, autophagy has been proved to facilitate hepatic lipid metabolism and regulate energetic homeostasis in the liver [10].

Autophagy is an evolutionarily conserved cellular degradation process that delivering cytoplasmic content to the lysosomes. Convincing evidence from rodent studies and clinical trials show that improving autophagy is a potential therapeutic strategy in hepatic disease [11]. In NAFLD, autophagy is profoundly reduced in hepatocytes and alters mitochondrial functions in the liver [12]. Importantly, recent studies demonstrate that dysregulation of hepatic autophagic flux is observed in the livers from patients with NAFLD [13]. Moreover, restored autophagy can help to eliminate these dysfunctional mitochondria, hence reduce the ROS level, and then potentially alleviate the NAFLD progression [7, 14]. Notably, many of the front-line therapies that improve NAFLD, including nonpharmacological caloric restriction, have also been shown to activate autophagy [15]. Hence, exploring a better therapeutic target on autophagy in NAFLD disease is needed.

High-temperature requirement protein A2 (HtrA2, also known as Omi) is a nuclear-encoded serine protease that resides in the intermembrane space of the mitochondria, and it can be released into the cytosol in response to various cellular stresses [16]. A recent study has reported that the serine protease HtrA2/Omi is a novel regulator of autophagy [16]. In vivo, a mature form of HtrA2/Omi can induce apoptosis by binding to the cytosolic inhibitor of apoptosis protein [17, 18]. Recently, studies have discovered that HtrA2/Omi inactivation does not cause early lethality in nonneuronal tissue. It has also been demonstrated that restoration of HtrA2/Omi expression can rescue CCl_4_-induced liver fibrosis and reverse mitochondrial dysfunction in hepatocyte [19]. Furthermore, increasing evidence has linked HtrA2/Omi to the pivotal cellular degradation process known as autophagy [20]. The microtubule-associated proteins 1A/1B light chain 3B (LC3I/LC3II) and SQSTM1/p62 are proteins that monitor autophagic activity [21]. HtrA2/Omi can induce autophagy through the degradation of Hax-1, a Bcl-2 family-related protein that suppresses autophagy in a Beclin-1-dependent pathway [20]. Full-length HtrA2/Omi enhances the turnover of LC3I to LC3II [20]. Importantly, inhibition of HtrA2/Omi results in abnormal autophagy and hepatic dysfunction in rats [22]. Therefore, HtrA2/Omi may be a new efficacious therapeutic target in improving mitochondrial dysfunction and autophagic disorder in NAFLD.

In this study, we reported for the first time that HtrA2/Omi played a protective role in NAFLD. To explore the effect of HtrA2/Omi, we increased HtrA2/Omi expression by adeno-associated virus and plasmid in vivo and in vitro, respectively. Moreover, the underlying mechanism was associated with HtrA2/Omi-mediated maintenance of hepatic mitochondrial homeostasis and autophagic ability. These findings suggested HtrA2/Omi could be a potential target for fatty liver disease therapy.

## Results

### Glucose tolerance, insulin resistance (IR), lipid metabolism disorder, and mitochondrial dysfunction were present in the mouse model of NAFLD

We induced a common model of NAFLD by feeding HFD for 13 weeks in C57BL/6 mice (Supplementary Fig. S1A). As shown in Supplementary Fig. S1B, HFD mice had a noticeable increase in bodyweight compared to the control group at W14. Moreover, no significant differences were observed in the average daily food intake examined at W14 (Supplementary Fig. S1C). HFD mice also showed large droplets, with moderate steatosis, ballooning, and inflammation compared to the control group (Supplementary Fig. S1D). The serum levels of ALT and AST in HFD mice, which reflect the degree of liver damage, were markedly increased compared to the controls (Supplementary Fig. S1E). And the serum TG, CHO, GLU, as well as hepatic TG, were also higher in HFD mice than those in control groups (Supplementary Fig. S1F, S1G). As the most critical metabolic organ, the liver plays an essential role in regulating blood-glucose homeostasis. In this study, HFD mice showed a significant increase in glucose tolerance and insulin resistance (Supplementary Fig. S1H, I).

Healthy mitochondria are crucial for the adequate control of energy metabolism in the liver. Accumulation of mitochondrial DNA (mtDNA) and impaired β-oxidation is an important hallmark of NAFLD progression [6]. Therefore, the mitochondrial function of liver tissue was analyzed. As shown in Supplementary Fig. S1J and S1K, we found that the mtDNA copy numbers and ATP levels were significantly reduced in the HFD model group compared to those of the normal control group. Moreover, HFD mice displayed a downregulated expression of genes involved in lipolysis and fatty acid oxidation in the liver (Supplementary Fig. S1L).

### Hepatic HtrA2/Omi expression, the rate-limiting enzyme of β-oxidation, and autophagy-associated proteins were decreased during NAFLD progression

**Fig. 1:**
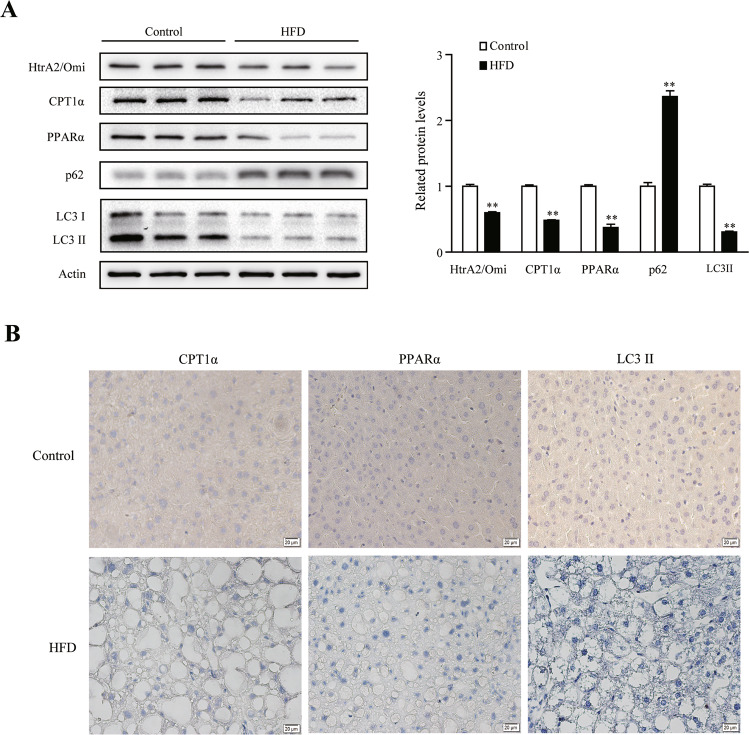
Effect of a high-fat diet on hepatic HtrA2/Omi expression, limiting enzyme of β-oxidation, and autophagy-associated proteins in the liver during NAFLD progression. **A** Western blot analysis of HtrA2/Omi, CPT1α, PPARα, p62, and LC3I/II in liver tissues. The histogram shows the ratio quantification of band intensities (*n* = 3). **B** Immunohistochemical staining of CPT1α, PPARα, and LC3II in the liver. scale bars: 20 μm (*n* = 6). Data are shown as means ± SEM. Student’s *t* test was used. **P < 0.01 vs. control group.

HtrA2/Omi has been reported to be involved in CCl_4_-induced liver fibrotic and age-related autophagic deficiency [3, 23]. In order to investigate whether alteration of HtrA2/Omi existed in hepatocyte in the progression of NAFLD, we validated the expression of HtrA2/Omi. Treatment with HFD for 13 weeks, compared to the chow diet group, hepatic HtrA2/Omi protein level, as well as protein level of CPT1α and PPARα, was significantly decreased (Fig. 1A, B). IHC and western blot revealed that the level of autophagy marker LC3II was significantly downregulated, and p62 increased in the livers of the HFD mice (Fig. 1A, B), suggesting that the autophagy was inhibited in HFD-induced NAFLD.

### Restoration of HtrA2/Omi expression attenuated HFD-induced hepatic steatosis

**Fig. 2:**
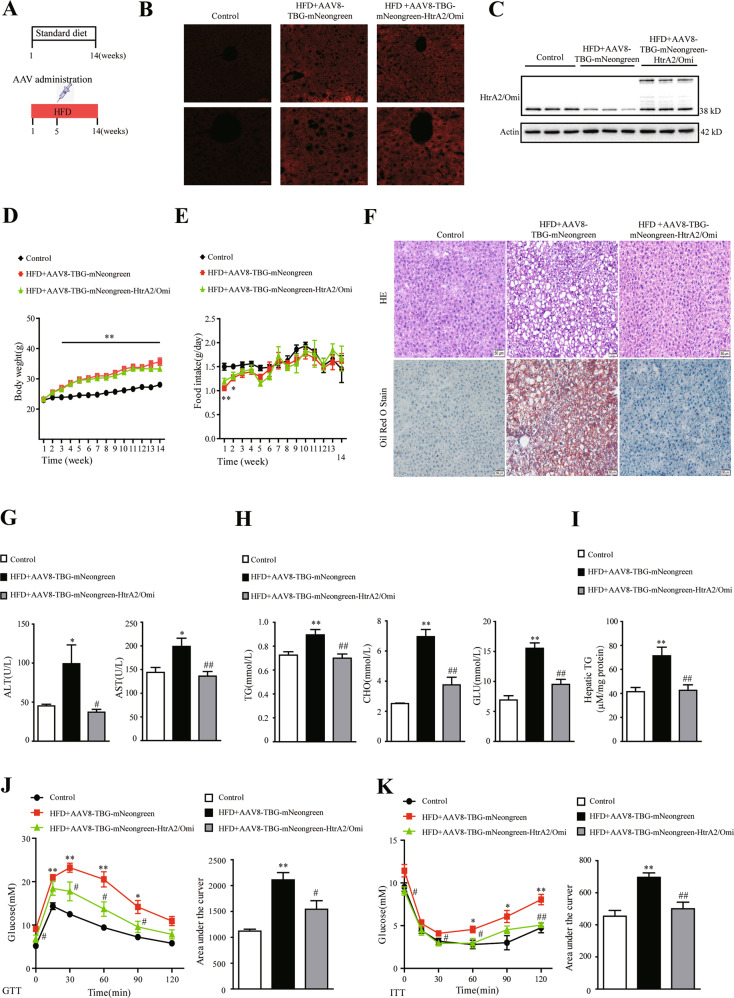
Expression of HtrA2/Omi improves metabolic parameters and decreases hepatic steatosis in HFD-fed obese mice. **A** Experiment design and time course. Groups were established as follows: Control mice (*n* = 7), HFD + AAV8-TBG-mNeongreen mice (*n* = 7) and HFD + AAV8-TBG-mNeongreen-HtrA2/Omi mice (*n* = 7). **B** The mNeongreen expression of liver tissues was observed under a fluorescent microscope, scale bars: 50 μm (up), 20 μm (down) (*n* = 6). **C** Western blot analysis of HtrA2/Omi in liver homogenates from the three groups (*n* = 3). **D** Bodyweight was monitored from W1 to W14 (*n* = 7). **E** Food intake from W1 to W14 (*n* = 7). **F** H&E staining of liver histology (up) and Oil Red O staining of liver lipid accumulation (down) at W14, scale bars: 50 μm (*n* = 7). **G** Comparisons of ALT and AST serum levels in treatment groups (*n* = 7). **H** The serum levels of lipid in different groups (*n* = 7). **I** Analyses of hepatic TG levels in mice (*n* = 7). **J** Measurement of plasma glucose during GTT (left); the AUC for GTT was calculated (right) (*n* = 7). **K** Blood-glucose levels (left) and the area under the curve (right) for glucose during ITT (*n* = 7). Data are shown as means ± SEM. One-way ANOVA was used. *P < 0.05 vs. control group; **P < 0.01 vs. control group; ^#^*P* < 0.05 vs. HFD + AAV8-TBG-mNeongreen group; ^##^*P* < 0.01 vs. HFD + AAV8-TBG-mNeongreen group.

To fully understand the effect of HtrA2/Omi on hepatic steatosis and related metabolic disorders, we further set up hepatic HtrA2/Omi-overexpressed mice models through tail-vein injections of AAV8-TBG-mNeongreen-HtrA2/Omi (Fig. 2A). AAV8-TBG serotype is highly hepatocyte-specific and has minimal off-target effects [24]. Tail-vein injection of AAV8-TBG-mNeongreen-HtrA2/Omi remarkable enhanced hepatic HtrA2/Omi expression in the liver of HFD group mice (Fig. 2B, C). As shown in Fig. 2D, E, there had no difference in body weights and food intake between the HFD + AAV8-TBG-mNeongreen group and HFD + AAV8-TBG-mNeongreen-HtrA2/Omi group at W14. As expected, HtrA2/Omi-overexpressed mice demonstrated normal liver histological characteristics, with a significant reduced of steatosis, ballooning, and inflammation compared with the HFD + AAV8-TBG-mNeongreen group (Fig. 2F). The NAS score showed a similar conclusion (Supplementary Fig. S2A). In biochemical analysis, HtrA2/Omi injection attenuated the HFD-induced significant increase of serum ALT and AST (Fig. 2G). Otherwise, HFD + AAV8-TBG-mNeongreen group exhibited elevated serum TG, CHO, and GLU levels, as well as hepatic TG contents, however, restoration of HtrA2/Omi improved serum and hepatic lipid profiles (Fig. 2H, I). In addition, we performed glucose-tolerance tests (GTT) and insulin-tolerance tests (ITT) to analyze the effect of hepatocyte HtrA2/Omi overexpression on blood-glucose homeostasis. HFD + AAV8-TBG-mNeongreen mice exhibited impairment of glucose tolerance and insulin resistance, while HtrA2/Omi-injected mice displayed lower blood glucose (Fig. 2J, K).

### Hepatic HtrA2/Omi overexpression improved mitochondrial dysfunction, hepatic fatty acid β-oxidation, autophagy, and energy expenditure

**Fig. 3:**
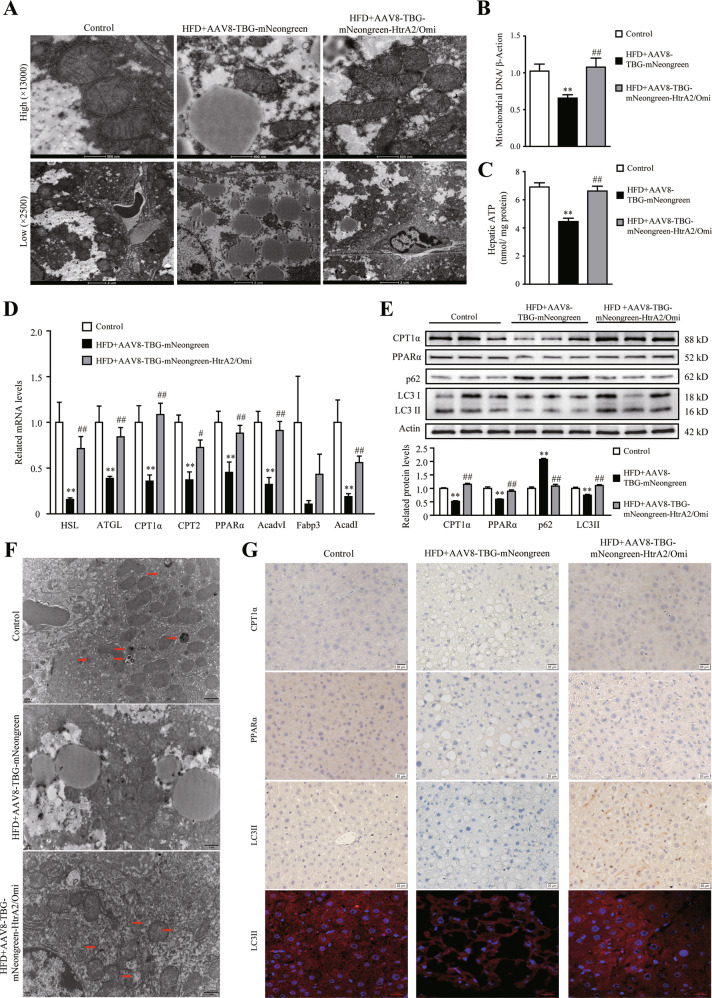
Hepatic HtrA2/Omi expression protects against mitochondrial impairment and enhances autophagy activity in fatty liver. **A** Representative TEM images of hepatic mitochondria. Mitochondrial morphology and integrity were observed. Scale bar (up): 0.5 μm; scale bar (down): 2 μm (*n* = 7). **B** Hepatic mtDNA content in different groups (*n* = 7). **C** The levels of ATP in the liver (*n* = 7). **D** The expression of genes involved in lipid metabolism in the livers (*n* = 7). **E** Western blot analysis of CPT1α, PPARα, p62, and LC3I/II in liver tissues. The histogram shows the ratio quantification of band intensities (*n* = 3). **F** Images from TEM show characteristic autophagosomes (red arrows) in hepatocytes. scale bar: 1 μm (*n* = 7). **G** Immunohistochemical staining of CPT1α, PPARα, and LC3II in the liver, scale bars: 20 μm (*n* = 6); immunofluorescence of LC3 puncta, scale bars: 10 μm (*n* = 6). Data are shown as means ± SEM. One-way ANOVA was used. **P < 0.01 vs. control group; ^#^*P* < 0.05 vs. HFD + AAV8-TBG-mNeongreen group; ^##^*P* < 0.01 vs. HFD + AAV8-TBG-mNeongreen group.

**Fig. 4:**
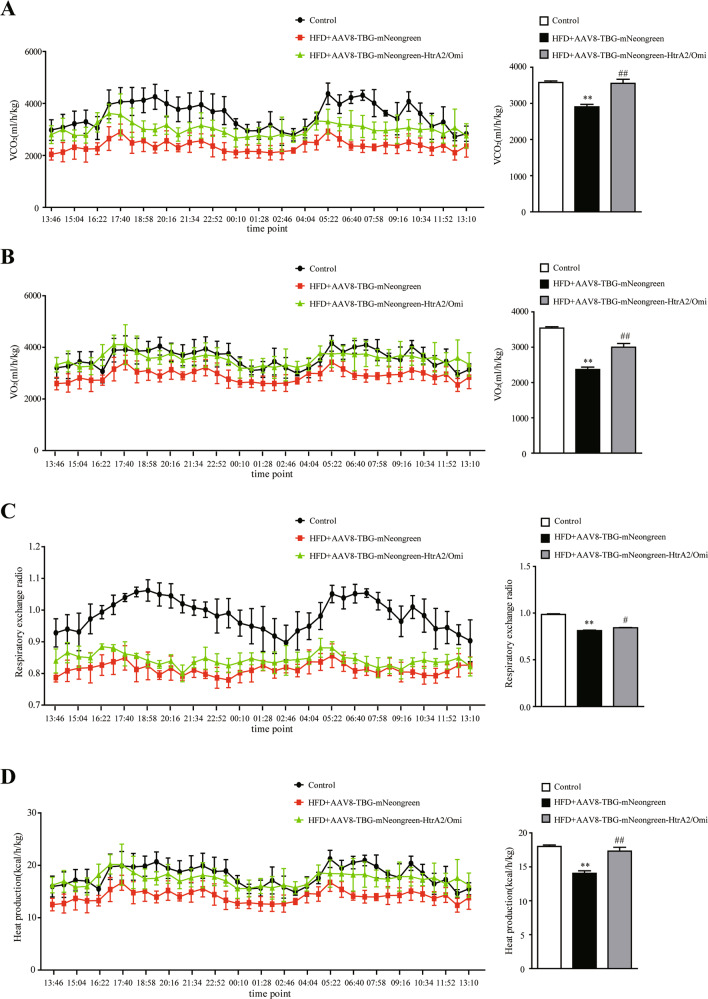
Indirect calorimetry analysis showing hepatic HtrA2/Omi enhances respiratory exchange ratio (RER) and heat production in NAFLD mice. **A** The CO_2_ production (VCO_2_) during 12-h dark/12-h light cycles measured in a metabolic cage (left) and the average VCO_2_ values were calculated (right) (*n* = 6). **B** The oxygen consumption (VO_2_) during two 12-h dark/12-h light cycles measured in a metabolic cage (left) and the average VO_2_ values were calculated (*n* = 6). **C** The RER measured in a metabolic cage (left) and the average RER (right) was calculated (*n* = 6). **D** The heat production measured in a metabolic cage (left) and the average values (right) was calculated (*n* = 6). Data are shown as means ± SEM. One-way ANOVA was used. *P < 0.05 vs. control group; **P < 0.01 vs. control group; ^##^*P* < 0.01 vs. HFD + AAV8-TBG-mNeongreen group.

To investigate whether HtrA2/Omi expression could reverse mitochondrial dysfunction, we used TEM to analyze ultrastructural changes. The TEM results showed that the mitochondria were scarce, swollen, and reveal disarranged cristae in HFD + AAV8-TBG-mNeongreen group. Conversely, HtrA2/Omi injection markedly improved mitochondrial structure, complying with the less swollen and relatively more organized mitochondrial cristae (Fig. 3A). Moreover, liver mtDNA copy numbers and ATP content were lower in HFD + AAV8-TBG-mNeongreen than control mice, while the HtrA2/Omi injection was able to improve this phenomenon (Fig. 3B, C). Otherwise, AAV8-TBG-mNeongreen-HtrA2/Omi treatment upregulated the expression of genes involved in fatty acid oxidation and lipolysis (Fig. 3D). Our results also showed that HtrA2/Omi expression elevated the protein levels of CPT1α and PPARα (Fig. 3E, G). Furthermore, a trend of reduced autophagic ability was observed in the HFD + AAV8-TBG-mNeongreen mice compared with the control group. However, AAV8-TBG-mNeongreen-HtrA2/Omi-treated mice restored autophagy activity, which was supported by elevated protein expression of LC3II and decreased p62 levels (Fig. 3E). In TEM analysis, the number of autophagic vacuoles around the lipid droplet was significantly reduced in the HFD + AAV8-TBG-mNeongreen group, while the HtrA2/Omi injection increased the numbers of autophagosomes (Fig. 3F). Furthermore, IHC staining demonstrated that LC3II protein level was increased in livers of the HFD + AAV8-TBG-mNeongreen-HtrA2/Omi group compared to HFD + AAV8-TBG-mNeongreen mice (Fig. 3G). Next, by using immunofluorescence, HtrA2/Omi-overexpressed mice exhibited an increased number of LC3 puncta, which indicated HtrA2/Omi promoted the initiation of autophagy (Fig. 3G). These data confirmed that hepatic HtrA2/Omi overexpression elevated autophagy in the liver. The liver is a key metabolic organ that governs the energy metabolism of the whole body [25, 26]. Using indirect calorimetry, we investigated if the improvement of liver mitochondrial function contributed to the systemic energy expenditure. The results revealed that VCO_2_, VO_2_, RER, and heat production of HFD + AAV8-TBG-mNeongreen mice were all lower than those of the control group, indicating the energy expenditure was reduced. Enhanced HtrA2/Omi expression in the liver resulted in the increased levels of VCO_2_, VO_2_, RER, and heat production (Fig. 4).

### FFA led to mitochondrial dysfunction, autophagic inhibition, and significant downregulation of HtrA2/Omi in vitro

**Fig. 5:**
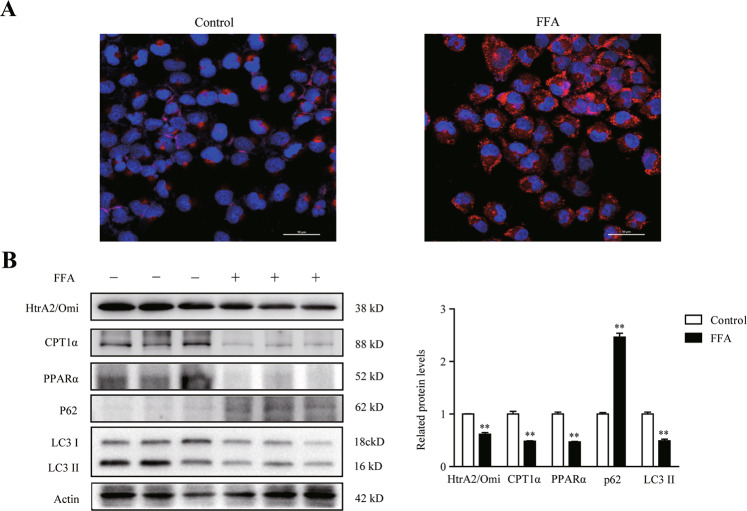
Effect of FFA on HtrA2/Omi expression, limiting enzyme of β-oxidation, and autophagy-associated proteins in vitro model of NAFLD. **A** Nile red staining of cells, scale bars: 50 μm (*n* = 3). **B** Western blot analysis of HtrA2/Omi, CPT1α, PPARα, p62, and LC3I/II in different groups. The histogram shows the ratio quantification of band intensities (*n* = 3). Data are shown as means ± SEM. Student’s *t* test was used. **P < 0.01 vs. control group.

As shown in Fig. 5A and Supplementary Fig. S3A, L02 cells stimulated with FFA in vitro showed a visibly raised intracellular lipid accumulation than control. In addition, the TG level in the FFA-treated cells was higher than that of the control cells (Supplementary Fig. S3B). Moreover, mtDNA levels obviously decreased after treatment with FFA, indicating that FFA impairs mitochondrial function (Supplementary Fig. S3C). Cellular ATP concentration was also reduced by fatty acid treatment (Supplementary Fig. S3D). Mitochondrial membrane potential (MMP) is regarded as drive force of oxidative phosphorylation in mitochondria [27]. A significant reduction of MMP was observed in FFA-induced cells (Supplementary Fig. S3E). As mitochondria are the major producers of ROS in cells [14], FFA induced a higher level of total ROS in L02 cells (Supplementary Fig. S3F). More importantly, MitoSOX™ staining confirmed that FFA stimulation resulted in a robust increase in mitochondrial ROS (Supplementary Fig. S3G).

Consist with the results in vivo, the protein level of HtrA2/Omi in the L02 with FFA-treatment was decreased (Fig. 5B). Otherwise, the protein levels of CPT1α and PPARα were also reduced (Fig. 5B). The protein level of LC3II was decreased, while the expression of p62 was enhanced (Fig. 5B). The results showed that FFA-treated cells exhibited impaired autophagy activity.

### HtrA2/Omi upregulation improved lipid metabolism in L02 cells, restored mitochondrial function, and activated autophagic flux

**Fig. 6:**
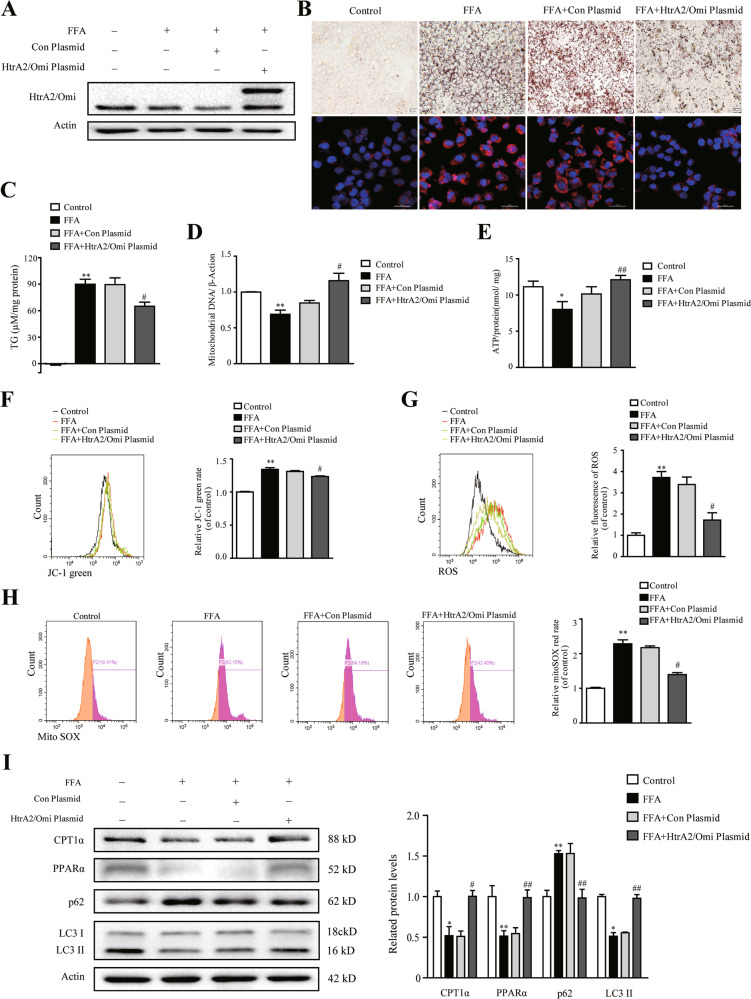
Elevated HtrA2/Omi expression promoted the improvement of lipid metabolism through restored mitochondrial function and activated autophagic flux in vitro. **A** The L02 transfect with human HTRA2 (NM_013247) pcDNA3.1-3xFlag-C plasmid was as HtrA2/Omi-overexpressed group. The L02 cells transfected with control plasmid was as vector control. Both the control and transfected L02 cells were treated with FFA. The protein level of HtrA2/Omi was assessed by western blot (*n* = 3). **B** Oil red O staining (up) and Nile red staining (down) of cells, scale bars: 50 μm (*n* = 3). **C** The levels of intracellular TG were measured (*n* = 3). **D** The mtDNA content of different groups was detected (*n* = 3). **E** Comparison of the ATP levels in different groups (*n* = 3). **F** The MMP of different groups was analyzed (*n* = 3). **G** The total ROS was detected by flow cytometry (*n* = 3). **H** Mitochondrial ROS in cells was assessed via MitoSOX™ Red dye by flow cytometry (*n* = 3). **I** Western blot analysis of CPT1α, PPARα, p62, and LC3I/II in different groups. The histogram shows the ratio quantification of band intensities (*n* = 3). Data are shown as means ± SEM. One-way ANOVA was used. *P < 0.01 vs. control group; **P < 0.01 vs. control group; ^#^*P* < 0.05 vs. FFA group; ^##^*P* < 0.01 vs. FFA group.

To further elucidate the effects of HtrA2/Omi on mitochondria and autophagy in vitro, HtrA2/Omi was overexpressed by plasmid (Fig. 6A). As shown in Fig. 6B, FFA-induced lipid accumulation was abrogated in L02 cells after HtrA2/Omi overexpression. And the level of TG in HtrA2/Omi-overexpressed cells was lower compared with FFA group (Fig. 6C). Moreover, the FFA-induced decrease of mtDNA was prevented by HtrA2/Omi overexpression (Fig. 6D). Similarly, ATP concentration was markedly increased after HtrA2/Omi upregulation (Fig. 6E). Furthermore, the collapse of MMP was significantly restored after treatment with HtrA2/Omi‐overexpressing plasmid (Fig. 6F). Abnormal ROS accumulation was diminished after HtrA2/Omi plasmid transfection (Fig. 6G). And the transfection with HtrA2/Omi‐expressing vector also decreased the production of mitochondrial ROS in vitro (Fig. 6H). In addition, HtrA2/Omi expression increased the protein level of CPT1α and PPARα after FFA exposure (Fig. 6I). Besides, HtrA2/Omi-transfection ameliorated autophagic inhibition, confirming by increased the LC3II protein level and promoted p62 degradation (Fig. 6I).

## Discussion

In this study, we demonstrated that HtrA2/Omi decreased in the fatty liver, and restoration of HtrA2/Omi reduced hepatic steatosis. The mechanism was associated with HtrA2/Omi-mediated enhancing mitochondrial function and autophagic flux.

NAFLD is becoming the most common chronic liver disease, and its pathogenesis involves many factors, including genetic factors, environmental factors, and metabolic factors. Recent data suggest that the accumulation of hepatic fat, which eventually leads to insulin resistance, is followed by the secondary effects comprising mitochondrial dysfunction and oxidative stress [28]. And the abnormal mitochondrial shape and mitochondrial dysfunction observed in HtrA2/Omi-deficient liver tissue are closely linked to liver fibrogenesis [19]. HTRA2 mutant mice exist increasing mtDNA damage and mtROS levels [19]. While it is unclear whether HtrA2/Omi changes during NAFLD progression. Our results showed that hepatic HtrA2/Omi was decreased in the livers of HFD mice compared with control mice.

Previous studies suggest that mitochondrial dysfunction is one of the key prevalent etiological factors of NAFLD. Mitochondria are vital intracellular organelles that are altered in response to cellular stress and metabolic changes in hepatocytes. When FFA is overloaded, the lipid storage in the liver exerts considerable stress on metabolism and subsequently causes lipotoxicity. The lipotoxicity markedly reduces the efficiency of the respiratory transport chain by producing ROS and endoplasmic reticulum stress. In addition, excessive accumulation of intracellular FFA also destroys the permeabilization of mitochondrial membrane and dephosphorylation capacity, as well as ATP production and antioxidant ability [29]. Moreover, rodent and other primate studies suggest that metabolic causes of NAFLD include reduction of hepatic mitochondrial function [30]. The overexpression of HtrA2/Omi in the liver protects against CCl_4_ induced liver fibrosis through modulating mitochondrial homeostasis in hepatocytes [19]. In this study, we investigated the effects of hepatic HtrA2/Omi on NAFLD by using AAVs. AAVs have become a focus of attention in the preclinical study, and provided a maintain effectiveness at the long term in the preclinical model [31]. AAV8-TBG serotype is highly hepatocyte-specific, and negligible expressed in extrahepatic tissues, meanwhile, tail-vein injection of AAV8-TBG does not influence the general health of mice [24]. Thus, here we used AAV8-TBG serotype which efficiently and targets hepatocytes to determine the effects of HtrA2/Omi on NAFLD. In this study, AAV8-mediated HtrA2/Omi-expressing mice under HFD significantly reversed hepatosteatosis, improved hepatic glucose tolerance, and insulin sensitivity. Otherwise, the restoration of hepatic HtrA2/Omi yielded a significant improvement of hepatic mitochondrial dysfunction. These results indicated that preserving mitochondrial function is an important mechanism underlying the effect of HtrA2/Omi on relieving NAFLD.

A recent study suggests that HtrA2/Omi regulates autophagy and inflammasome activation by preventing the prolonged accumulation of the inflammasome adapter [16]. And one characteristic of NAFLD is the suppression of autophagy in the liver [32]. Impaired autophagic flux occurs in livers from both patients and mammalian models, and in FFA-loaded human hepatocytes [33]. Improved autophagy by genetic or chemical treatments can alleviate liver steatosis in animal models of NAFLD [34]. In this article, we examined the autophagy status by using TEM. In nonalcoholic hepatic steatosis, autophagic vesicle-like structures are decreased, and the numbers of autophagsomes are increased, indicating that the improvement of autophagic ability [35]. Consist with previous studies, autophagic vacuoles were less observed in the fatty livers of HFD + AAV8-TBG-mNeongreen mice, but increased in the HFD + AAV8-TBG-mNeongreen-HtrA2/Omi groups. This indicated hepatic HtrA2/Omi overexpression increased autophagic ability. On the other hand, LC3II has been proved to be a major coordinators marker reliably associated with completed autophagosomes, and activation of the autophagic flux causes an increase in the expression of LC3II [36, 37]. Moreover, induction of the LC3 puncta indicates that the initiation of autophagy, which suggests autophagosomes are directly involved in LDs capture [38]. p62 is a selective substrate for autophagy that recognizes ubiquitinated cargo and links with autophagosomes, and accumulation of p62 leads to a decline in autophagic flux [36, 37]. In our study, HtrA2/Omi expression increased LC3II, promoted the initiation of LC3 puncta, and decreased p62 expression in vivo, indicating a recovery of the autophagic flux. In HtrA2/Omi‐expressing vector-treated cells, the blocked autophagic activity was also restored. These data supported that hepatic HtrA2/Omi expression improved autophagic inhibition in NAFLD.

Liver tissue plays an important role in systemic metabolic processes [39]. Notably, we further demonstrated whether localized improvement in hepatocyte mitochondrial function was adequate to alter whole-body energy expenditure. Interestingly, compared to HFD + AAV8-TBG-mNeongreen group, hepatic HtrA2/Omi-expressing mice exhibited elevated energy expenditure. These results may be associated with the brown adipose tissue (BAT)-mediated thermogenesis. A previous study shows that brown adipose tissue plays a major role in upregulating the basal metabolic rate, and has been implicated in metabolism and energy expenditure [40]. Activation of BAT has the capacity to divert lipid from the liver and thus ameliorate NAFLD [41, 42]. Otherwise, the effects of adipose tissue on weight and metabolic regulation depend on the different window of developmental stages. The changes of bodyweight and metabolism are not always synchronized. In our study [43], dietary intervention of different time points (at the beginning of the postweaning, puberty, or post-puberty periods) could improve metabolic dysregulation in postnatal overfed rats, but only the treatment during the postweaning period could markedly reverse obesity (including body and adipose tissue weight). Given that the development of adipose tissue is phased, different time points could lead to different effects. Here, we just observed hepatic HtrA2/Omi expression led to improvement of energy expenditure, but the importance of BAT activation was not assessed in this study, which need to be assessed extensively. Meanwhile, studies have shown that liver tissue contributes to 20–30% of whole-body energy expenditure [39], and the effects of HtrA2/Omi expression on whole-body energy expenditure may be associated with the improvement of hepatocyte mitochondrial function.

In summary, we reported that HtrA2/Omi expression could greatly reduce liver steatosis, improve glucose tolerance and hepatic insulin resistance in a mouse model of HFD-induced NAFLD. Furthermore, the molecular mechanisms were involved in the restoration of mitochondrial dysfunction and autophagic activity in hepatocytes. Altogether, our results pointed toward HtrA2/Omi as a potential therapeutic target against NAFLD.

## Materials and methods

### Animals and diets

Six-week-old male C57BL/6J mice were purchased from the Laboratory Animal Center of Nanjing Medical University. All mice were kept in a specific pathogen-free facility under controlled light (06:00 am–06:00 pm) and temperature (22 ± 2 °C) conditions. After 1 week, the mice with similar body weights were divided randomly. Mice were fed a standard chow diet (ND, 10% of energy as fat; D12450B; Research Diets, New Brunswick, NJ, USA) or a HFD (high-fat diet, 60% of energy as fat; D12492; Research Diets, New Brunswick, NJ, USA) for 13 weeks. HFD chow had blue dye which is different from the ND chow. All animal experiments were performed according to the protocols approved by the Institutional Animal Care and Use Committee of Nanjing Medical University (IACUC 2011009). The sample size was chosen based on the prior study [44].

### Adeno-associated virus preparation and injection

The C-terminal mNeongreen-3Xflag tagged HtrA2/Omi-expression plasmid was created by Beijing SyngenTech Co. Ltd. (Beijing, China). AAV8-TBG-WPRE-SV40_polyA delivery vector construction, viral packaging, and titration were performed by Beijing SyngenTech Co. Ltd. (Beijing, China). In a separate trial, 6-week-old male C57BL/6J mice divided into two groups to be fed a standard chow diet (ND, 10% of energy as fat; D12450B; Research Diets, New Brunswick, NJ, USA) or an HFD (high-fat diet, 60% of energy as fat; D12492; Research Diets, New Brunswick, NJ, USA). Previous studies showed that 4 weeks of HFD feeding could induce distinct hepatic steatosis [45, 46]. After 4 weeks of feeding, the HFD group was randomly sorted into two groups: (1) HFD + AAV8-TBG-mNeongreen mice group (HFD chow and AAV8-TBG-mNeongreen administration) and (2) HFD + AAV8-TBG-mNeongreen-HtrA2/Omi mice (HFD chow and AAV8-TBG-mNeongreen-HtrA2/Omi administration). These mice were injected with 5 × 10^11^ vector genome adenoma-associated virus expressing a control virus encoding the fluorescent protein (mNeongreen, AAV8-TBG-mNeongreen) or HtrA2/Omi (AAV8-TBG-mNeongreen-HtrA2/Omi).

### Cell culture and treatments

Human normal liver cell line L02 (ATCC, Shanghai, China) was cultured in a medium consisting of DMEM: 10% fetal bovine serum, 100 U/mL penicillin, and 100 μg/mL streptomycin. The cells were placed in a 5% CO_2_ humidified incubator at 37 °C. To establish an in vitro model of hepatic steatosis, L02 cells were stimulated with 1 mM FFA containing sodium oleate and sodium palmitate (2:1 at molar ratio) and 1% fatty acid-free bovine serum albumin (BSA) for 24 h. The L02 cells used in our research has been published [47].

### Overexpression of HtrA2/Omi in vitro

The pcDNA3.1-3xFlag plasmid harboring a human HtrA2/Omi gene (NM_013247) and control plasmid were purchased from Youbio Biological Technology Co., Ltd. (Changsha, China). Prior to DNA transfection, L02 cells were plated in a six-well plate and incubated overnight. On day 2, the plasmid (5 μg) was delivered to L02 cells using Lipofectamine 2000 (Thermo, USA) following the manufacturer’s instructions. After 4 h incubation, the transfection medium was replaced by an equal volume of cell growth medium. On day 3, L02 cells were treated with FFA for 24 h.

### Immunofluorescence (IF) and Nile red staining

After washing with PBS three times, frozen liver sections were blocked with 5% BSA for 1 h, and then incubated with primary antibodies overnight at 4 °C. After washing, sections were incubated with Cy3-labeled IgG secondary antibodies. Images were observed under a fluorescence microscope (Zeiss, Germany). The following primary antibodies were used: Anti-LC3II (L7543, sigma, Germany, 1:100). Nile red staining was performed by lab technicians from Servicebio Technology Co. Ltd. (Wuhan, China). Briefly, after being treated with 4% paraformaldehyde for 15 min, cells were incubated with dilute Nile red working solution for 10 min. Then cells were washed three times and incubated with DAPI. Images were observed under a fluorescence microscope (Nikon, Japan).

### Histological measurement and scoring

Livers were preserved in 4% paraformaldehyde solution, sliced, dehydrated, and embedded in paraffin. Liver sections (5 μm) were then stained with Hematoxylin and Eosin (H&E) staining. Oil Red O staining was performed by lab technicians from Servicebio Technology Co. Ltd. (Wuhan, China). Briefly, frozen liver sections were washed with 60% isopropanol, and then sections were stained in Oil Red solution for 10 min. After washing with 60% isopropanol, sections were stained with hematoxylin. Images were acquired using an Olympus microscope. The severity of NAFLD was assessed as in previous studies [41, 48].

### Immunohistochemistry (IHC) staining

The expressions of CPT1α, PPARα, and LC3II of the liver tissues were assessed as described in the previous study [41]. Briefly, after deparaffinization and rehydration, 8-μm-thick sections were incubated in 3% hydrogen peroxide for 10 min. Then these slides were blocked with 5% goat serum and then incubated with primary antibodies overnight at 4 °C. After washing with PBS, the sections were incubated with the corresponding secondary antibodies for 1 h at room temperature. And then streptavidin–HRP was added. Color development was performed with DAB Kits and hematoxylin. Images were observed under a microscope (Olympus BX51, Olympus, Tokyo, Japan). The following primary antibodies were used: anti-CPT1α (ab128568, Abcam, USA, 1:100). Anti-PPARα (YT3835, Immunoway, USA, 1:100); anti-LC3II (L7543, Sigma, Germany, 1:100).

### Glucose-tolerance tests (GTT) and insulin-tolerance tests (ITT)

For the glucose-tolerance tests (GTT), mice were intraperitoneal injection (i.p.) injected with d-glucose (2 g/kg) after 16-h fasting. For the insulin-tolerance test (ITT), mice were i.p. injected with insulin (1 U/kg) after 6-h fasting. Blood-glucose levels were measured respectively from the tail vein using a glucose monitor (OneTouch Ultra; On Call EZII, China) at 0, 15, 30, 60, 90, and 120 min.

### Transmission electron microscopy

Fresh livers (1 mm^3^) were collected and fixed for longer than 2 h in 2% paraformaldehyde and 2.5% glutaraldehyde solution and next rinsed thrice with 0.1 M PBS. The samples were then postfixed in 1% osmium tetroxide, dehydrated through an acetone gradient, and finally embedded. Semithin sections were sliced by an ultrathin slicing machine. The sections were stained with 3% lead citrate-uranium acetate. The ultrastructural morphological features were observed by a transmission electron microscope (FEI Tecnai G2 Spirit Bio TWIN, 120 kV).

### Serum and liver biochemical analyses

After collection, Liver samples homogenized and blood samples were centrifuged at 2000 rpm for 30 min and stored at −80 °C until analysis. The serum aspartate aminotransferase (AST), alanine aminotransferase (ALT), total cholesterol (CHO), triglyceride (TG), and glucose (GLU) were measured using an automatic biochemical analyzer (7100, Hitachi, Japan). Levels of hepatic lipid and ATP were estimated using commercially available kits were analyzed (A111-1, Nanjing Jiancheng Bioengineering Institute, Nanjing, China; S0026, Beyotime, China) according to the manufacturer’s instructions.

### Energy expenditure

The energy expenditure was monitored using the indirect calorimetry TSE PhenoMaster metabolic cages as described in the previous study [42]. Indirect calorimetry and locomotor activity monitoring system (TSE Phenomaster, TSE, Germany) were utilized to evaluate whole-body metabolic rate, including oxygen consumption (VO_2_), carbon dioxide production (VCO_2_), and heat production. At 13 weeks, mice were acclimated to the system for a 48 h adaptation period, and were free access to food and water. Data were collected during the next 48 h and then calculated the respiratory exchange ratio (RER) (VCO_2_/VO_2_).

### Western blot analysis

The whole tissue or cell lysates were prepared as mentioned [47]. An equal amount of protein samples was separated by 12% SDS-PAGE and transferred onto the PVDF membranes (Millipore, MA, USA). The membranes were blocked with 5% dry nonfat milk for 3 h at room temperature. Then the blots were hybridized with specific antibodies of HtrA2/Omi, LC3II/I, p62, and β-actin overnight at 4 °C After washing, blots were incubated with HRP-conjugated secondary antibody for 1 h at room temperature and then washed again with PBST. The immune complexes were detected using a chemiluminescence system (ChemiDoc XRS+, Bio-Rad, USA). The following primary antibodies were used: anti-HtrA2 (ab32092, Abcam, USA, 1:1000); anti-CPT1α (ab128568, Abcam, USA, 1:1000); anti-PPARα (YT3835, Immunoway, USA, 1:1000); anti-p62 (ab109012, Abcam, USA, 1:1000); anti-LC3II (L7543, Sigma, Germany, 1:1000); anti-β-actin (AP0060; Bioworld, China, 1:1000).

### Quantitative real-time PCR (RT-qPCR)

Total RNA was isolated with Trizol Reagent (TAKARA, Japan) according to the manufacturer’s instructions. The reverse-transcribed to cDNA was using the M-MLV reverse transcriptase (TAKARA, Japan). Quantitative PCR was performed using the SYBR Green master mix (Vazyme, China) following the manufacturer’s instructions. The mRNA level was determined using the 2^-ΔΔCT^ method with the CT values normalized using β-actin as an internal control. The sequence of all primers involved in this study is shown in Table 1.

**Table 1:** Primer sequences for real-time PCR (RT-PCR) analysis.

Primer	Forward	Reverse
HSL	ACTGAGATTGAGGTGCTGTC	AGGTGAGATGGTAACTGTGAG
ATGL	GTCACCAACACCAGCATCC	CGAAGTCCATCTCTGTAGCC
CPT1α	TGGCATCATCACTGGTGTGTT	TCTAGGGTCCGATTGATCTTTG
CPT2	CAGCACAGCATCGTACCCA	TCCCAATGCCGTTCTCAAAA
PPARα	AACATCGAGTGTCGAATATGTGG	CCGAATAGTTCGCCGAAAGAA
Acadvl	ACTACTGTGCTTCAGGGACAA	GCAAAGGACTTCGATTCTGCC
Fabp3	ACCTGGAAGCTAGTGGACAG	TGATGGTAGTAGGCTTGGTCAT
Acadl	TTTCCTCGGAGCATGACATTTT	GCCAGCTTTTTCCCAGACCT

### Flow cytometry assay

In brief, cells were cultured in a 12-well plate, and treated with various stimulations, then the cells were harvested and washed once with PBS. The cell pellet was incubated with a different reagent working solution for 20 min at 37 °C in the dark, and washed twice with cold PBS. The fluorescence intensity was detected using a flow cytometer (BECKMAN COULTER, America). ROS and JC-1 Assay kit purchased from Beyotime Institute of Biotechnology (Nanjing, Jiangsu, China) was used according to the manufacturer’s instructions. Mitochondrial ROS levels were detected using MitoSOXTM reagent.

### Statistics analysis

All Data were represented as mean ± SEM. The statistical significance of differences was determined using either the Student’s *t* test (two-tailed) or one-way ANOVA followed by a post hoc least significant difference (LSD) *t* test. A value of *P* < 0.05 was considered statistically significant.

## Supplementary information

Supplementary figure legends

Figure S1

Figure S2

Figure S3

Original Data File

## Data Availability

The datasets used and/or analyzed during this study are available through the corresponding author on reasonable request.
