# Supplementary material for: HtrA2/Omi mitigates NAFLD in high-fat-fed mice by ameliorating mitochondrial dysfunction and restoring autophagic flux
Source: Cell Death Discov. 2022 Apr 21;8:218. doi: 10.1038/s41420-022-01022-4 (PMC9023526; doi:10.1038/s41420-022-01022-4)
Supplement: Supplementary file 1 — Supplementary figure legends [file 41420_2022_1022_MOESM1_ESM.docx]

**Fig S1. Characterization of the mouse model of diet-induced NAFLD.** (A) Scheme of HFD-induced NAFLD progression. (B) Body weight in mice was monitored from W1 to W14 (n=7). (C) Food intake of control and HFD mice from W1 to W14 (n=7). (D) H&E staining of liver tissue (up) and Oil Red O staining of liver fat (down) at W14, scale bars: 50 μm (n=7). (E) The activity of ALT and AST in serum of different groups (n=7). (F) The serum levels of lipid in different groups (n=7). (G) The levels of TG in liver of different groups (n=7). (H) Blood glucose levels (left) and the area under the curve (right) for glucose during glucose tolerance test (GTT) (n=7). (I) Blood glucose levels (left) and the area under the curve (right) for glucose during insulin tolerance test (ITT) (n=7). (J) Hepatic mtDNA content in different groups at W14 (n=7). (K) The levels of ATP in liver (n=7). (L) The expression of genes involved in lipid metabolism in the livers (n=7). Data was shown as means ± SEM. Student’s t-test was used. * P < 0.05 vs. control group; ** P < 0.01 vs. control group.

**Fig S2. Liver NAS score at W14 in mice.** HFD+AAV8-TBG-mNeongreen mice exhibited steatosis, ballooning and inflammation (n=6). Data was shown as means ± SEM. One-way ANOVA was used. ** *P* < 0.01 vs. control group; ## *P* < 0.01 vs. HFD+AAV8-TBG-mNeongreen group.

**Fig S3. L02 cell steatosis and mitochondrial dysfunction after FFA treatment for 24 h.** (A) Oil red O staining of the control and FFA cells (n=3). (B) TG concentrations in the control and FFA-treated cells were measured (n=3). (C) Comparison of the mtDNA content in different groups (n=3). (D) The ATP content of different groups was analyzed (n=3). (E) Comparison of mitochondrial membrane potential (MMP) by using JC-1 staining (n=3). (F) The total ROS was detected by flow cytometry (n=3). (G) Mitochondrial ROS in cells was assessed via MitoSOX™ Red dye by flow cytometry (n=3). Data was shown as means ± SEM. Student’s t-test was used. * P < 0.05 vs. control group; ** P < 0.01 vs. control group.
