# Supplementary figures and images for: HtrA2/Omi mitigates NAFLD in high-fat-fed mice by ameliorating mitochondrial dysfunction and restoring autophagic flux
Source: Cell Death Discov. 2022 Apr 21;8:218. doi: 10.1038/s41420-022-01022-4 (PMC9023526; doi:10.1038/s41420-022-01022-4)

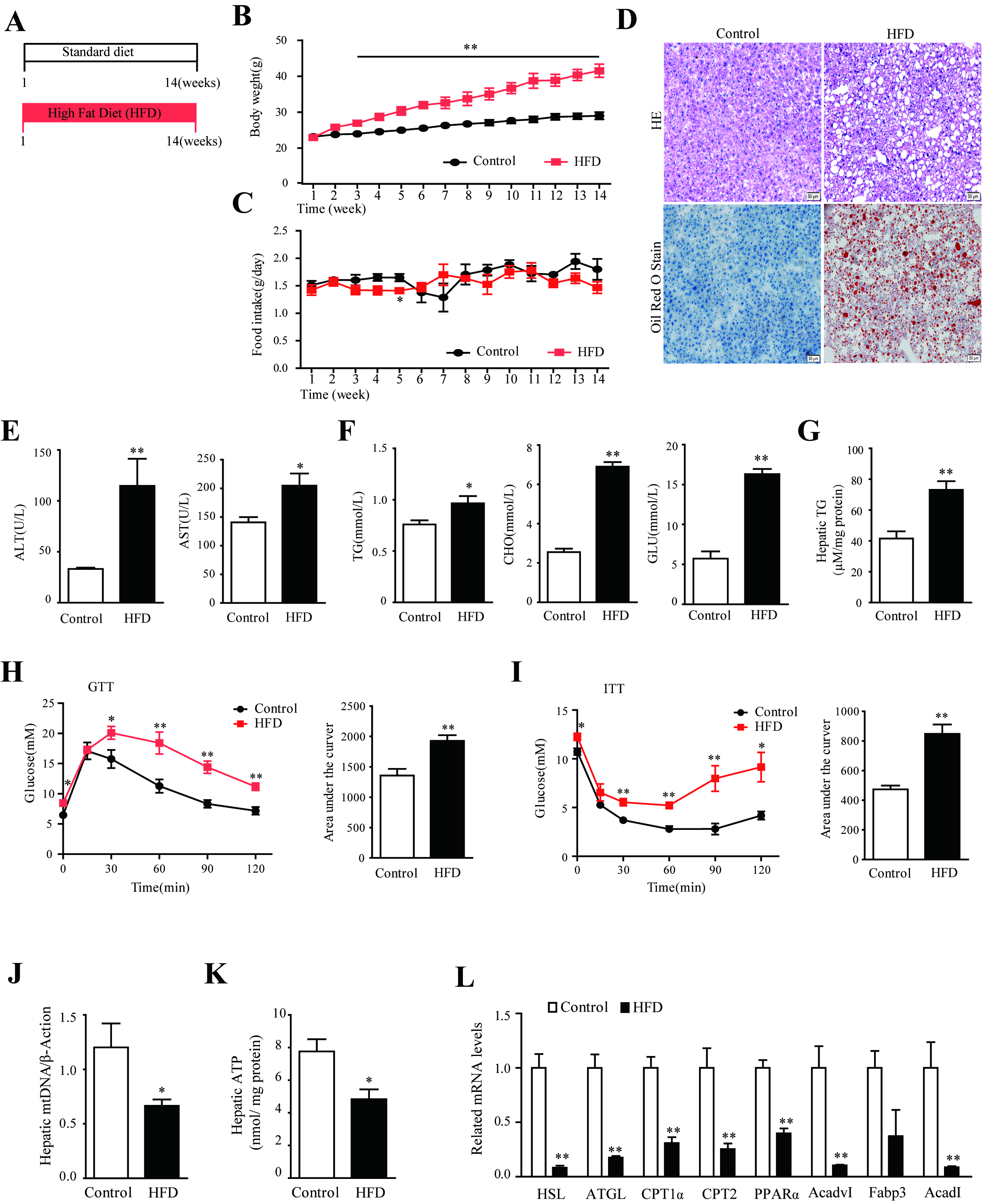

Supplement: Supplementary file 2 — Figure S1 [file 41420_2022_1022_MOESM2_ESM.tif]

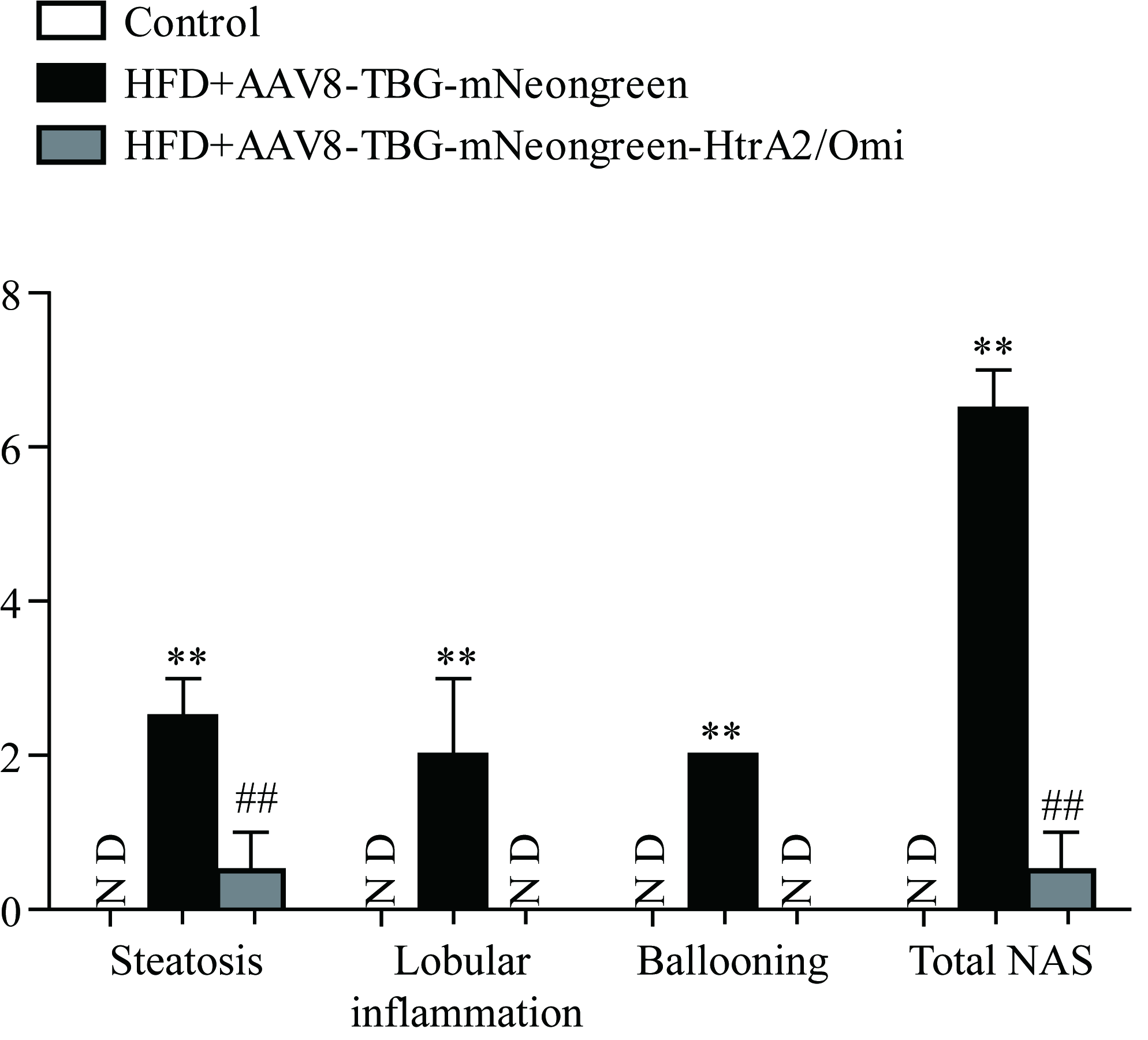

Supplement: Supplementary file 3 — Figure S2 [file 41420_2022_1022_MOESM3_ESM.tif]

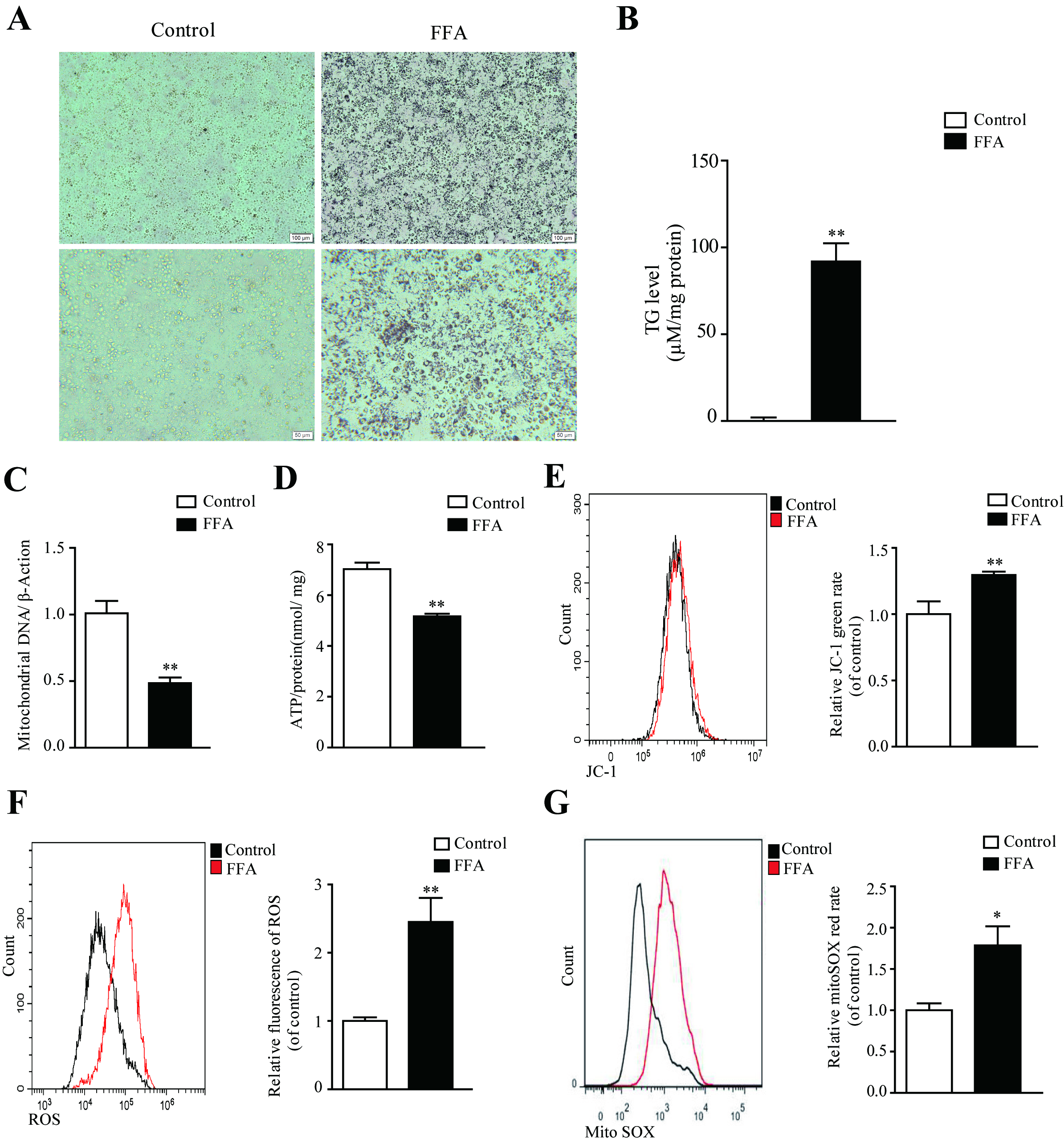

Supplement: Supplementary file 4 — Figure S3 [file 41420_2022_1022_MOESM4_ESM.tif]
