# Supplementary material for: HtrA2/Omi mitigates NAFLD in high-fat-fed mice by ameliorating mitochondrial dysfunction and restoring autophagic flux
Source: Cell Death Discov. 2022 Apr 21;8:218. doi: 10.1038/s41420-022-01022-4 (PMC9023526; doi:10.1038/s41420-022-01022-4)
Supplement: Supplementary file 5 — Original Data File [file 41420_2022_1022_MOESM5_ESM.docx]

**Figure 2-Actin
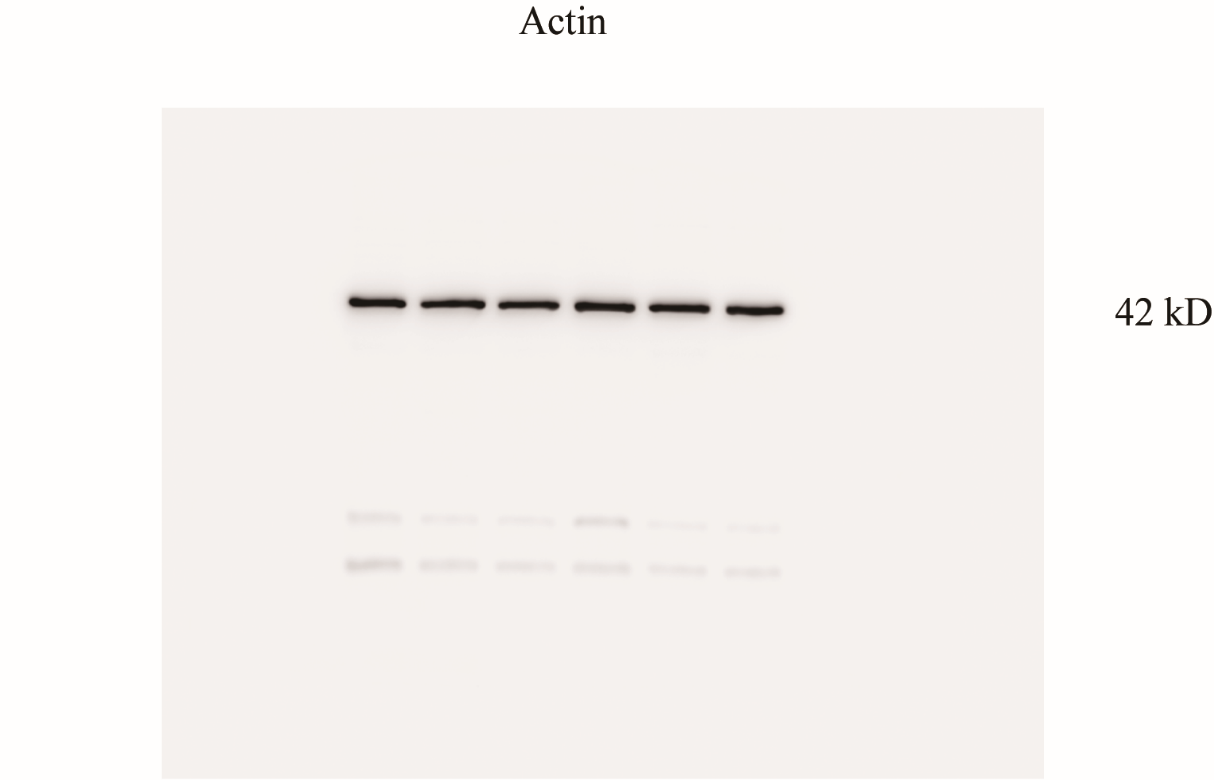
**


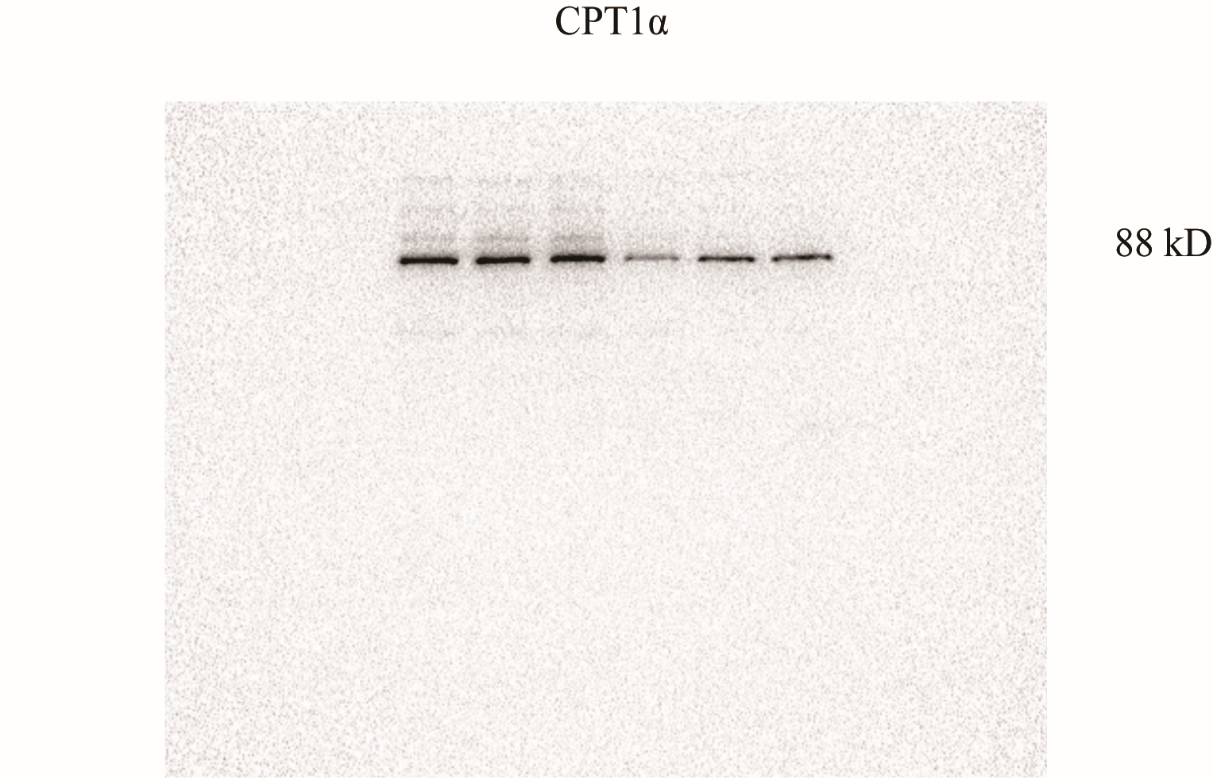


**Figure 2-CPT1α**


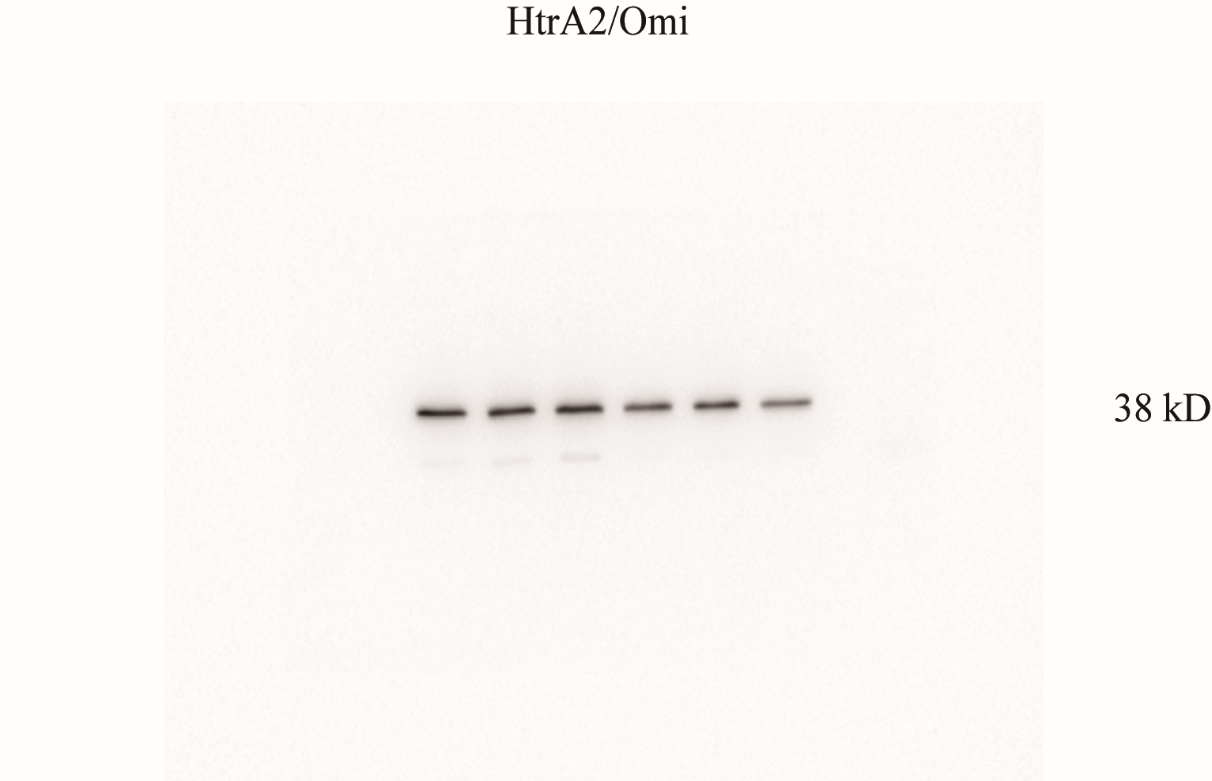


**Figure 2-HtrA2/Omi**

**Figure 2-LC3I and LC3II
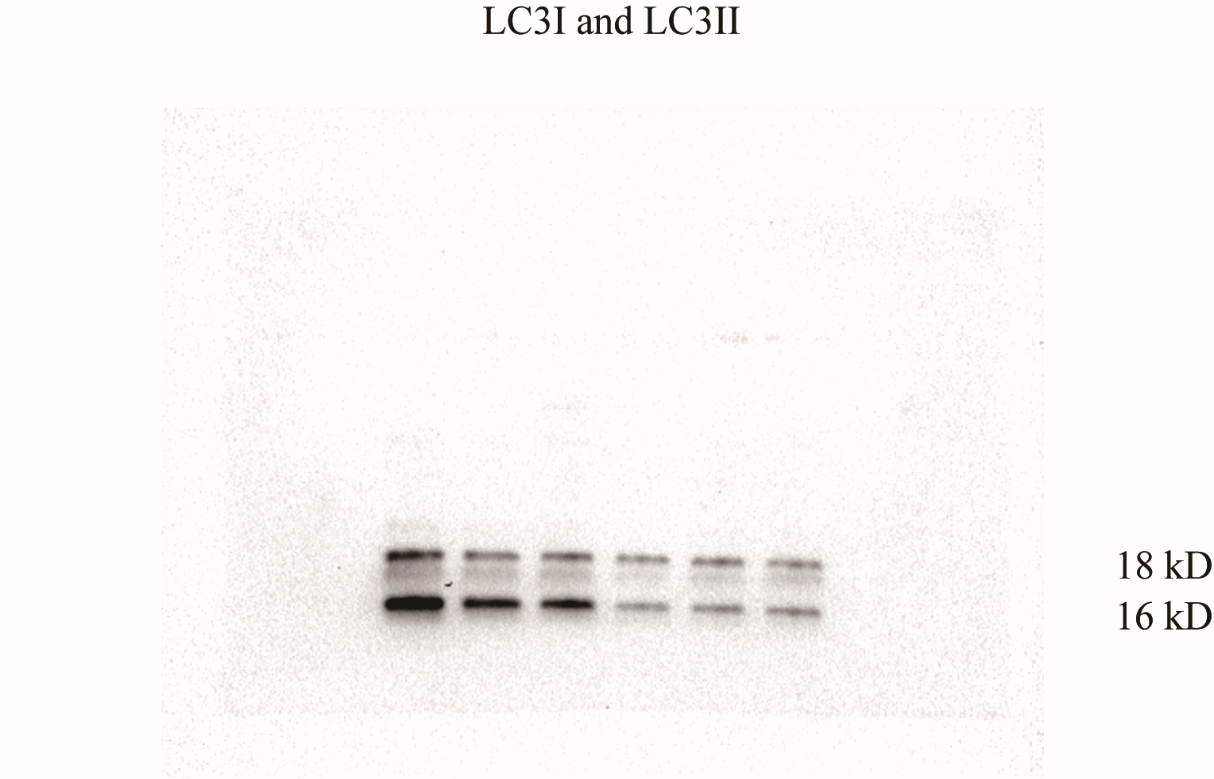
**

**Figure 2-p62
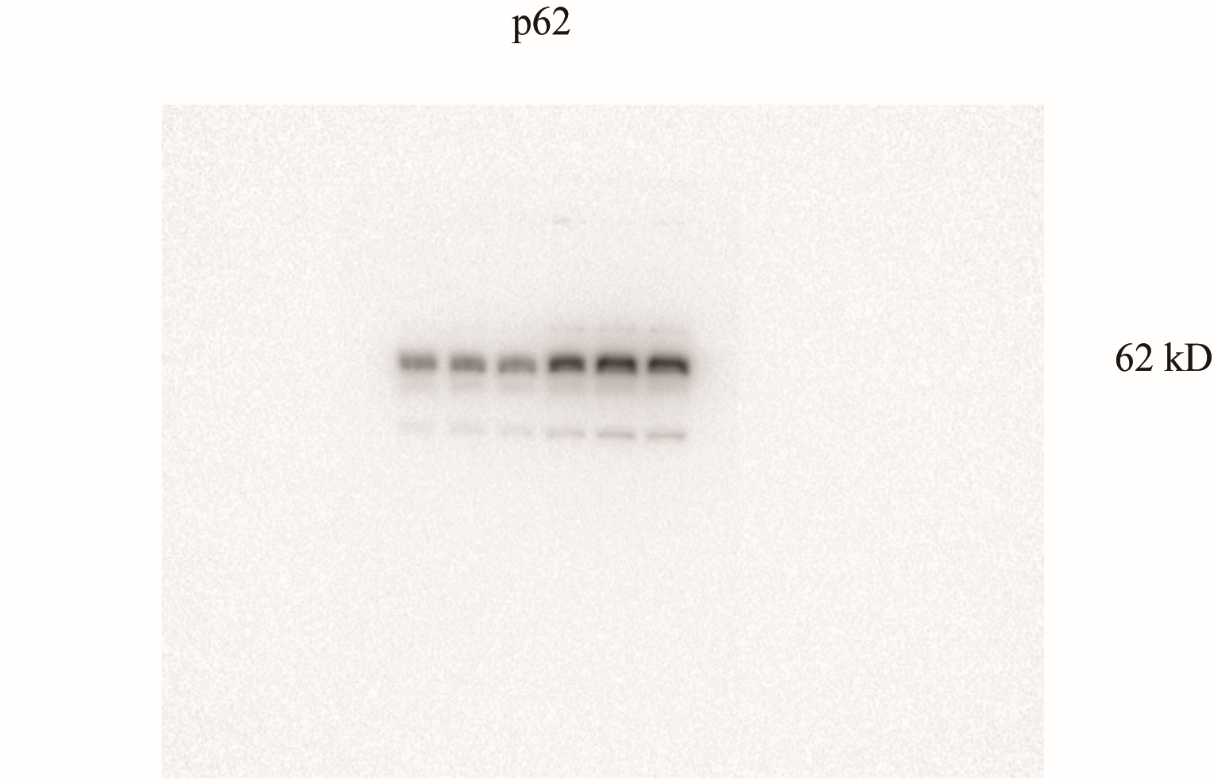
**

**Figure 2-PPARα
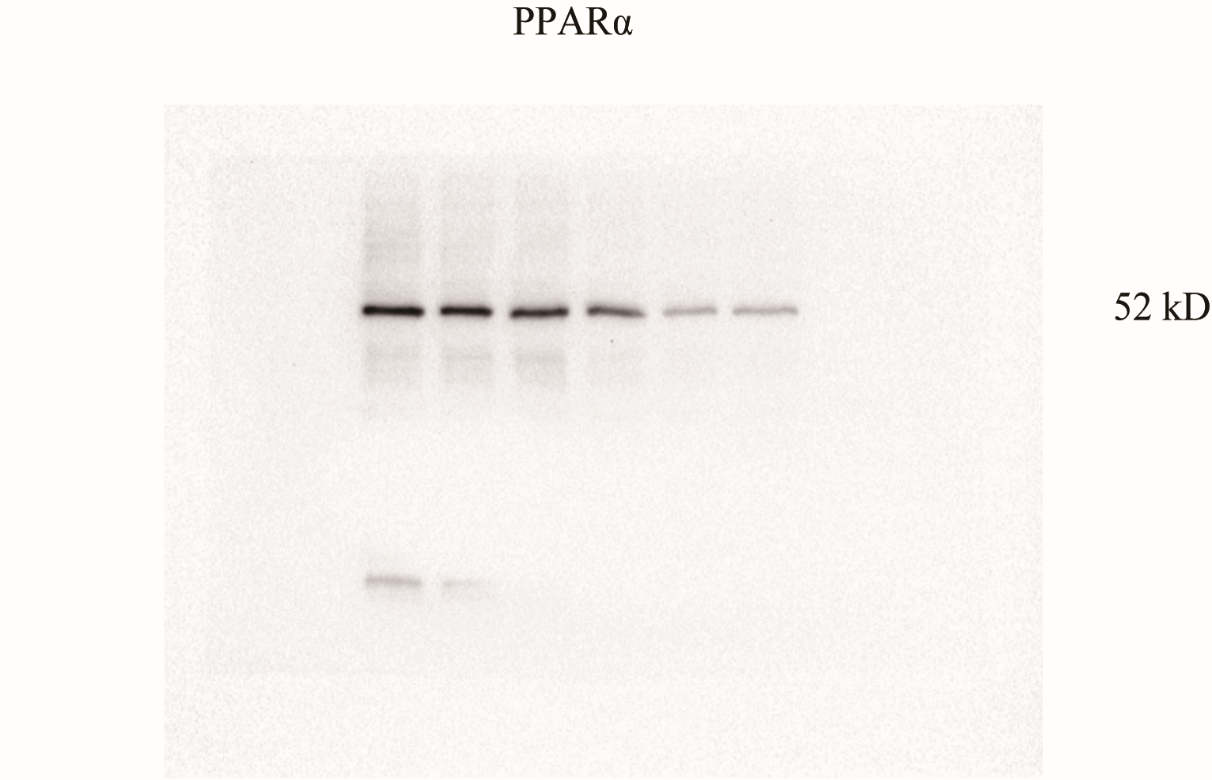
**

**Figure 3-Actin
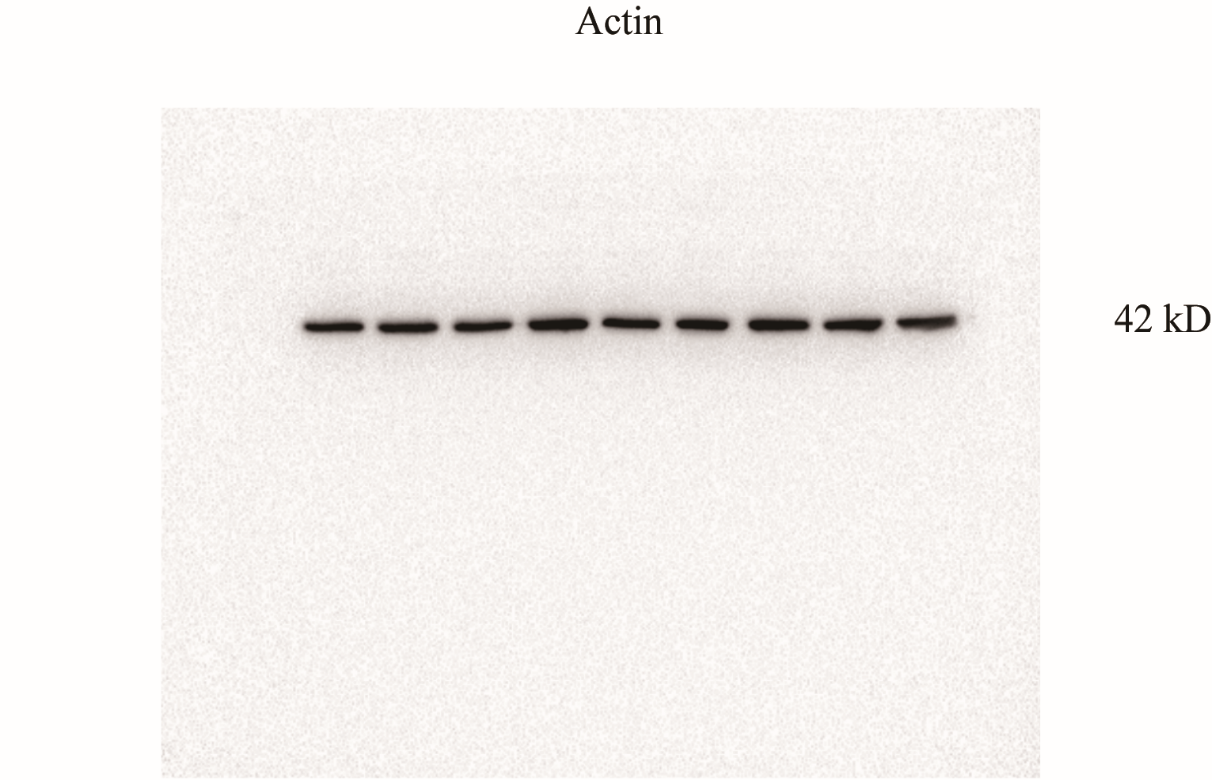
**

**Figure 3-HtrA2/Omi
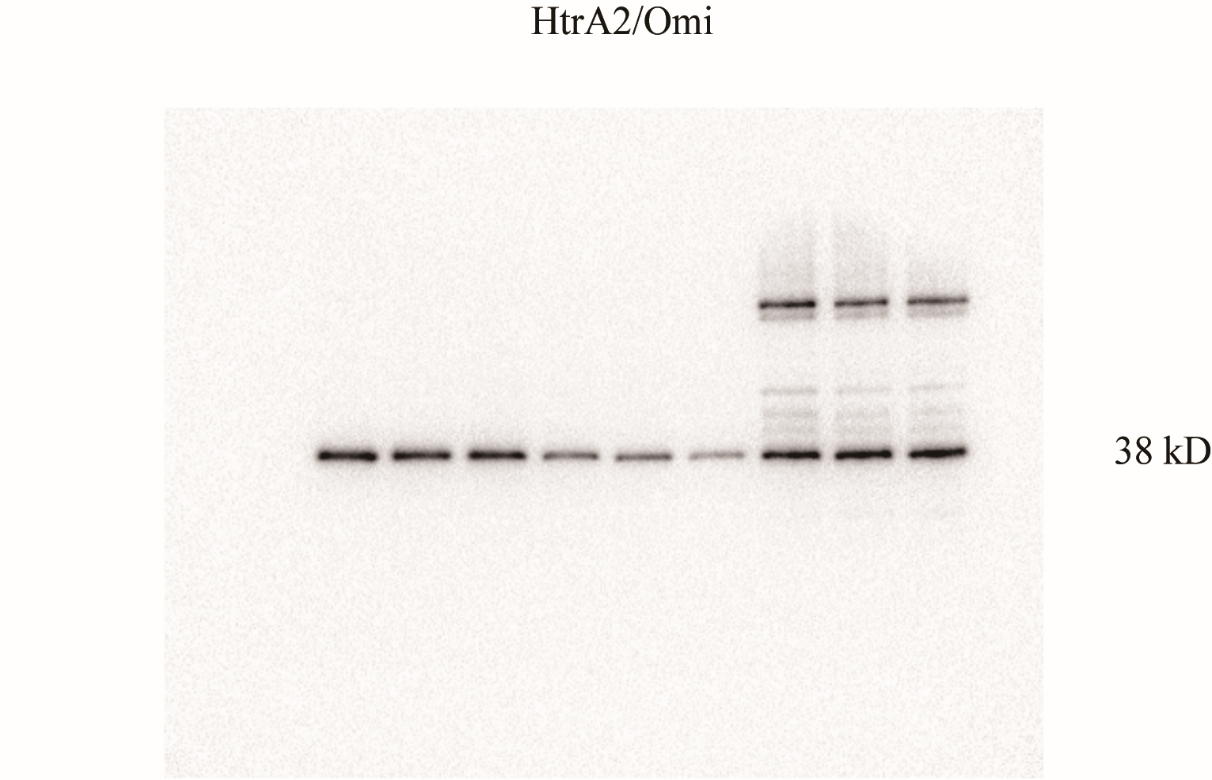
**

**Figure 4-Actin
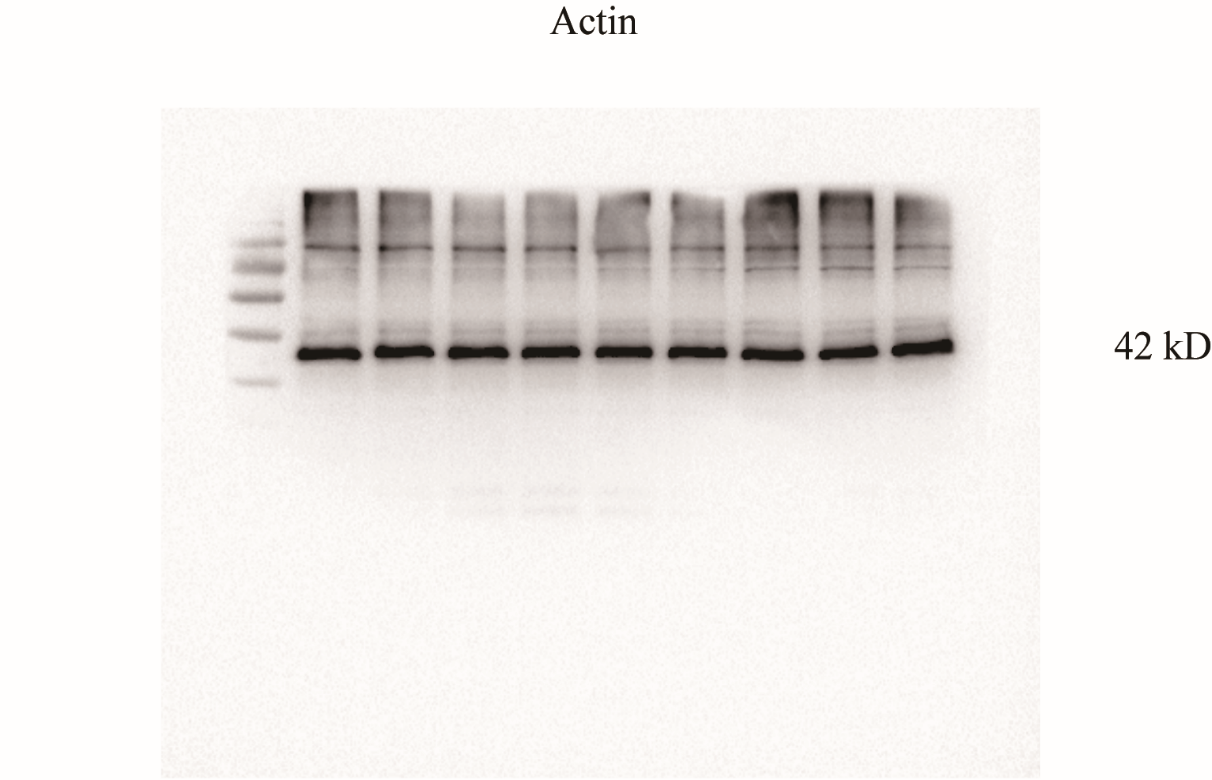
**

**Figure 4-CPT1α
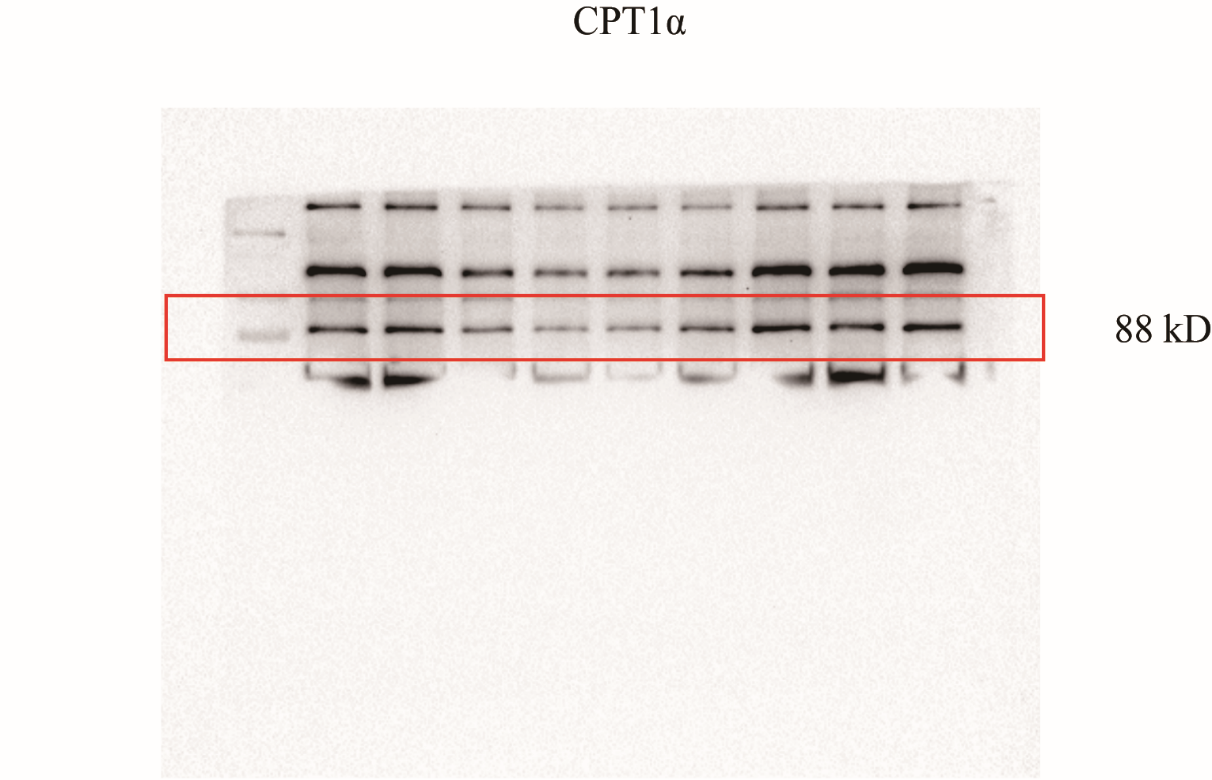
**

**Figure 4-LC3I and LC3II
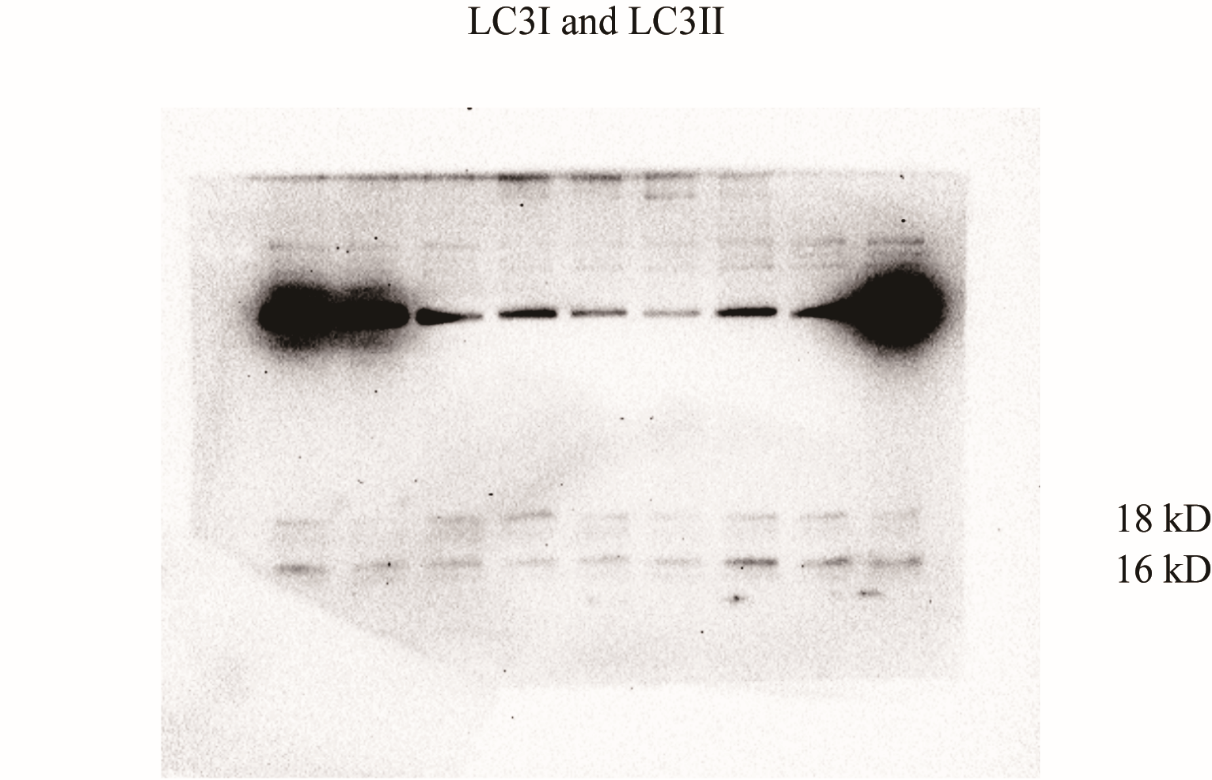
**

**Figure 4-p62
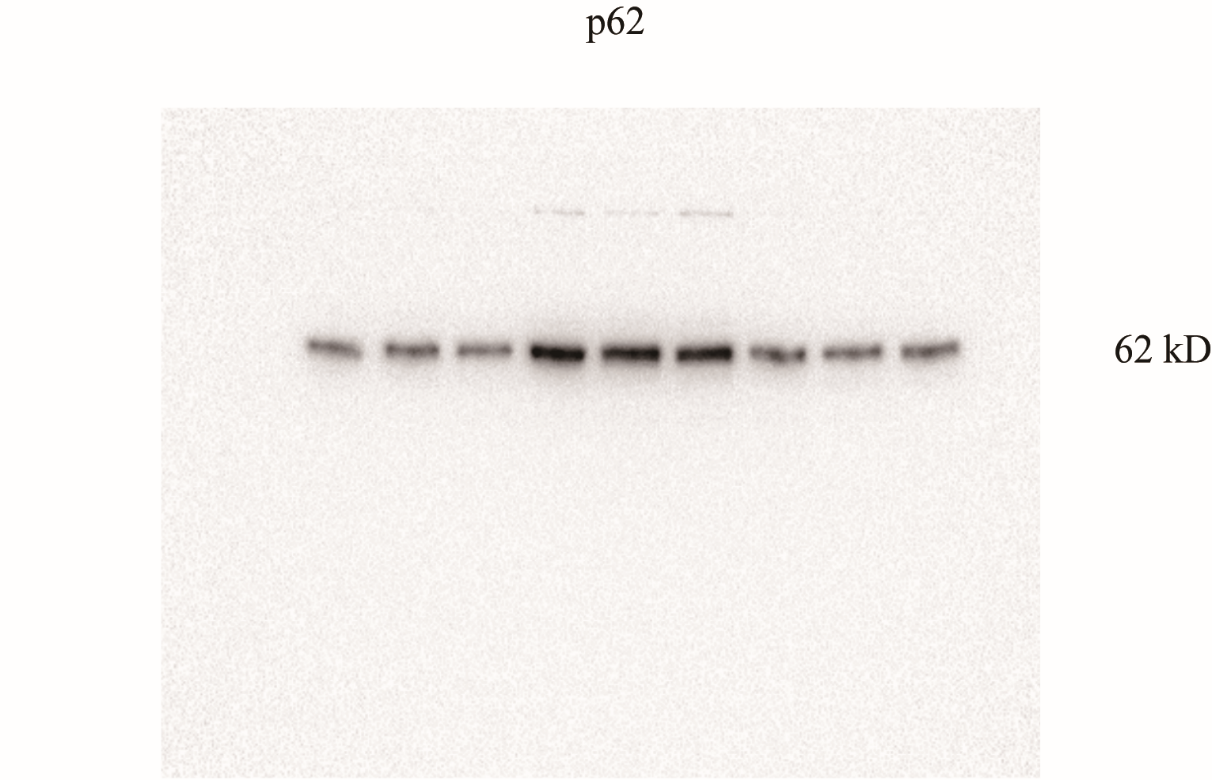
**

**Figure 4-PPARα
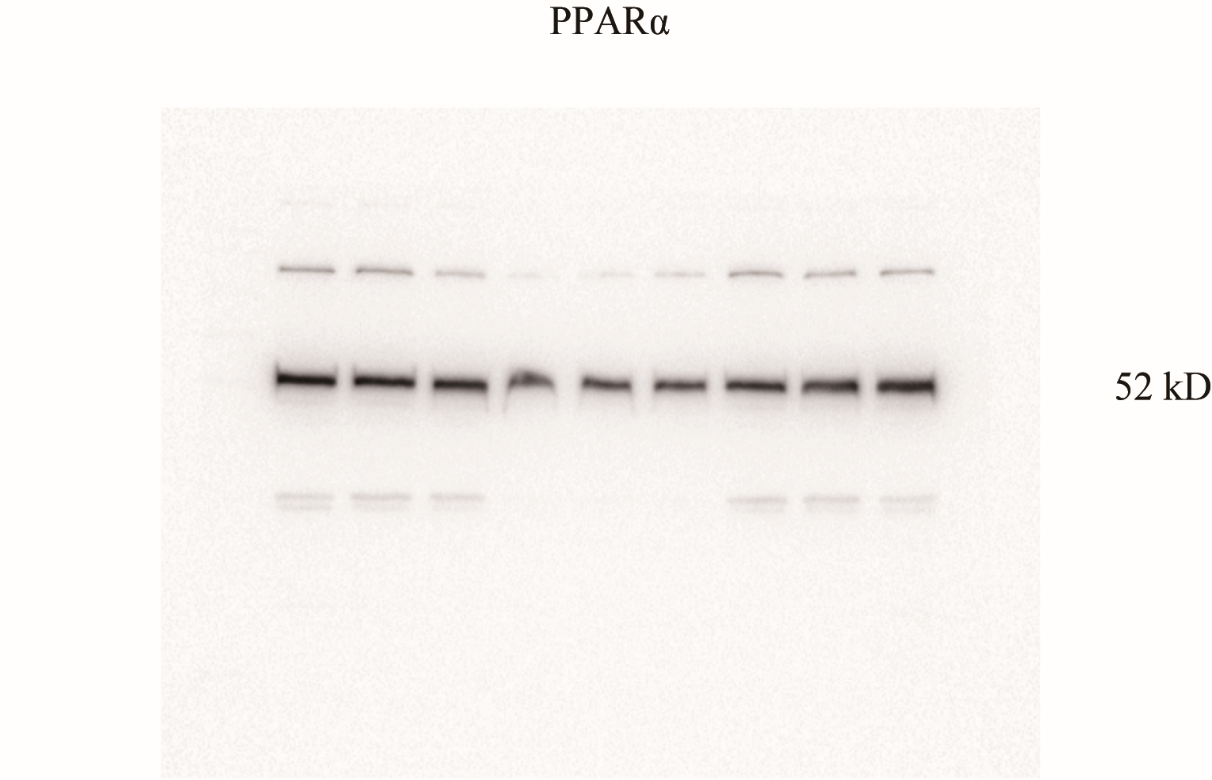
**

**Figure 6-Actin
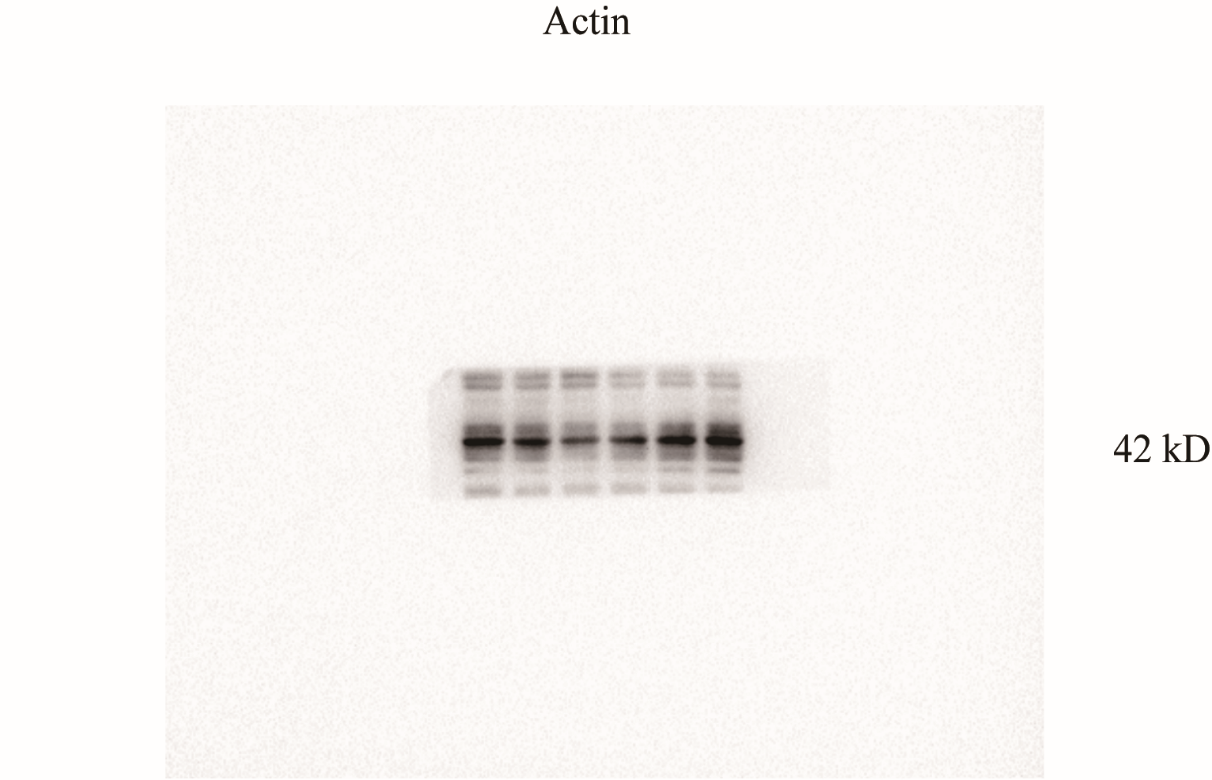
**

**Figure 6-CPT1α
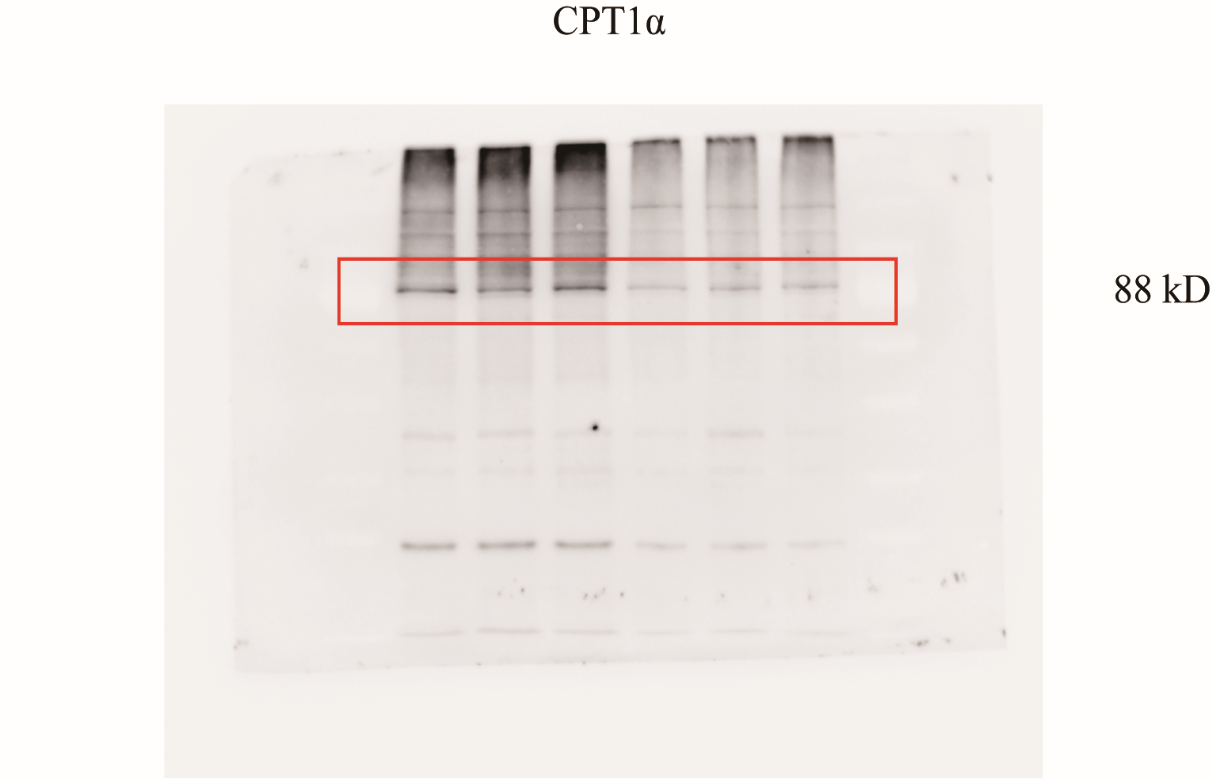
**

**Figure 6-HtrA2/Omi
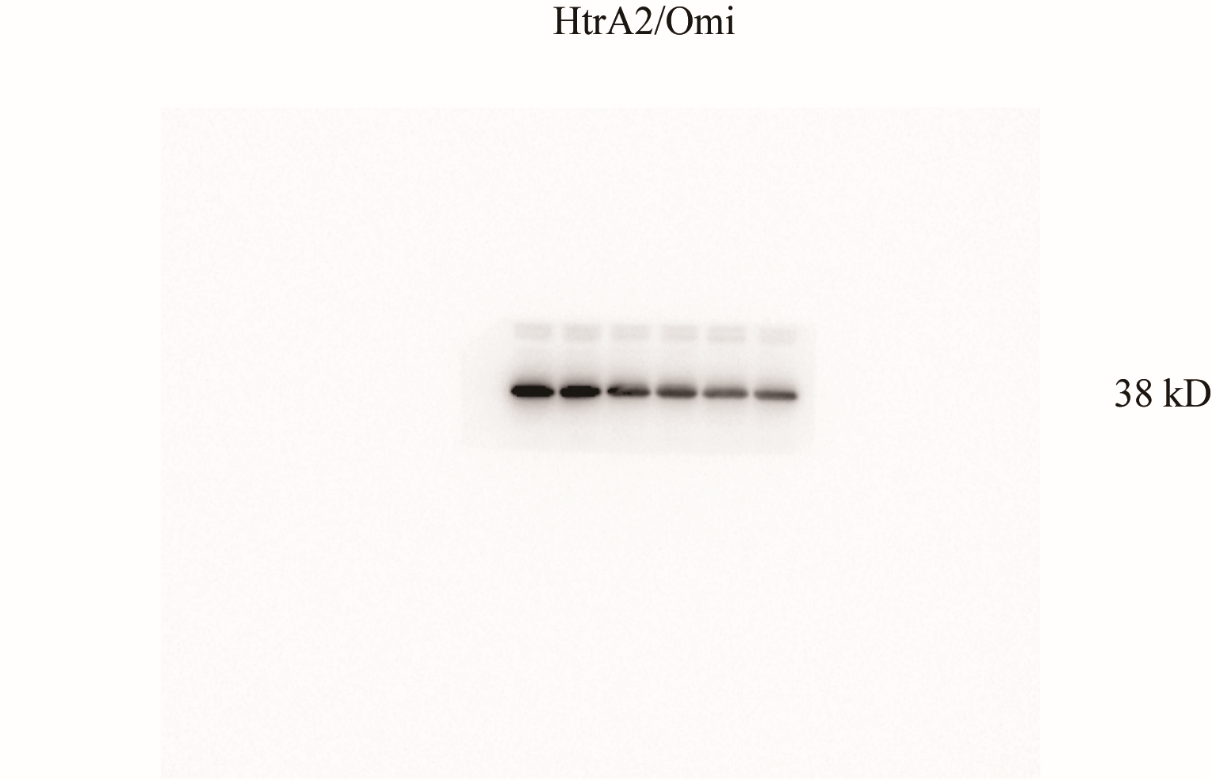
**

**Figure 6-LC3I and LC3II
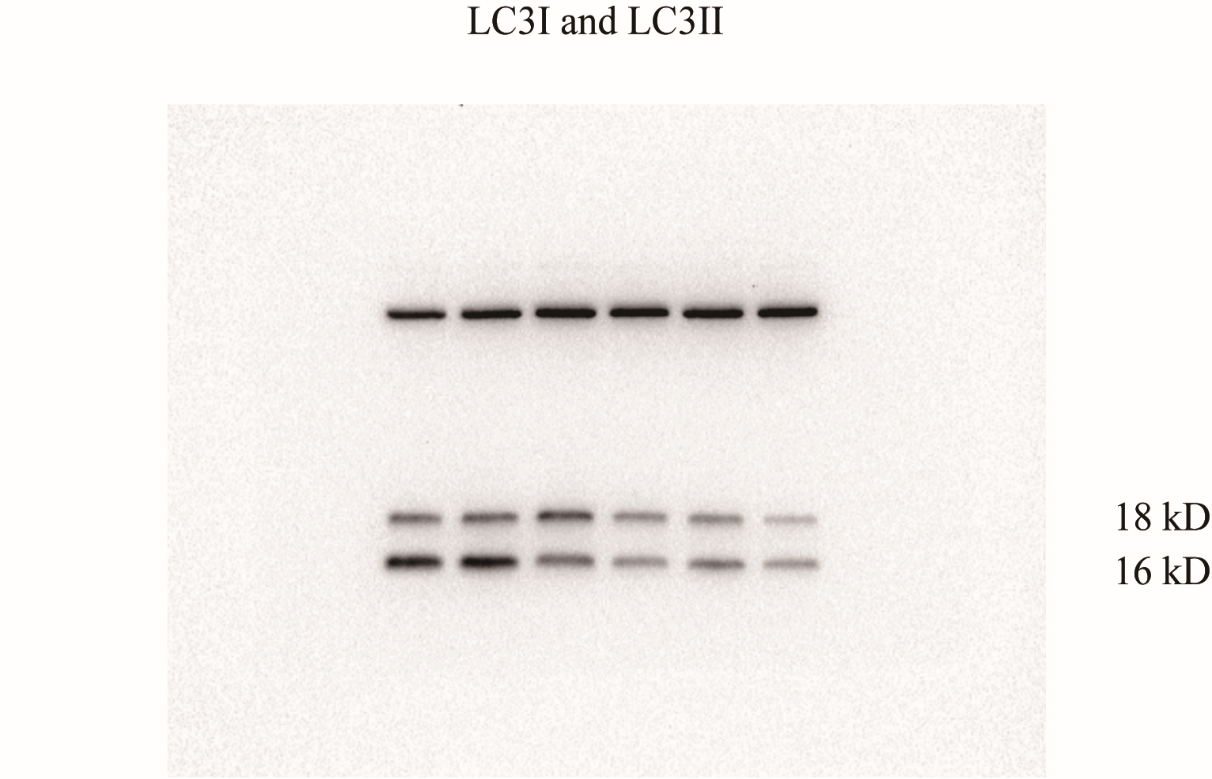
**

**Figure 6-p62
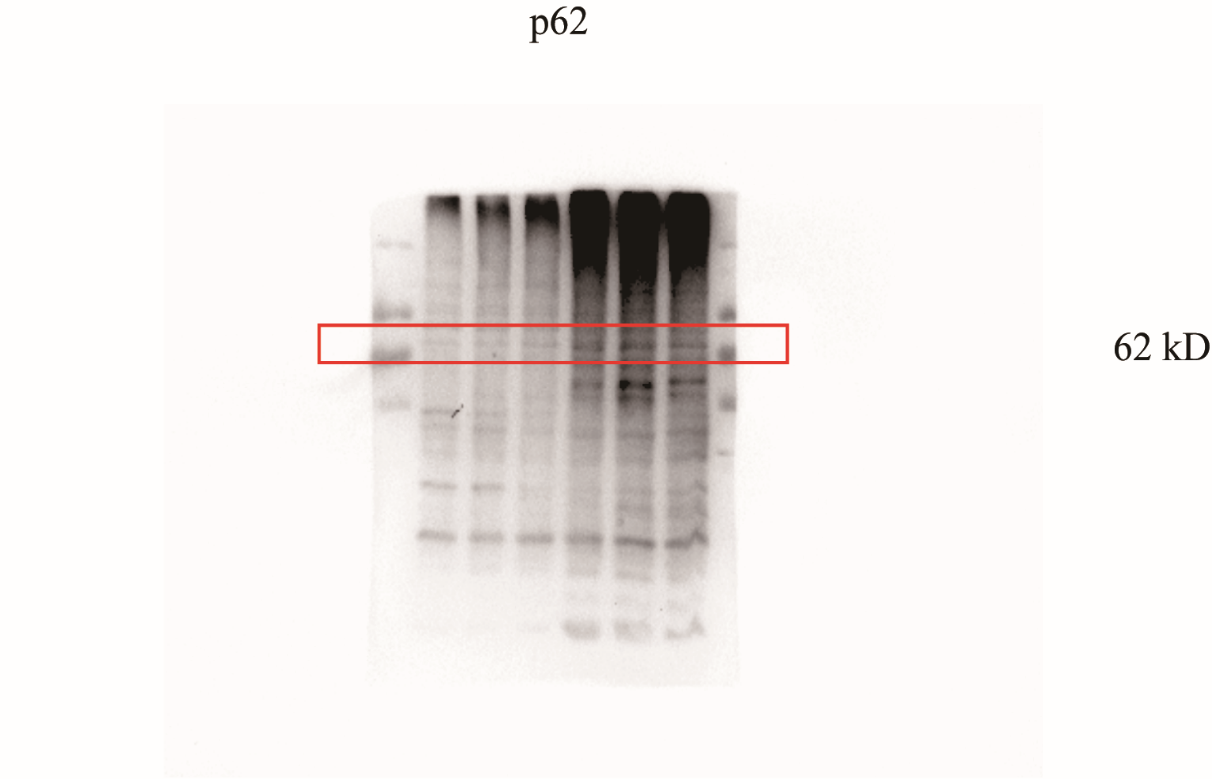
**


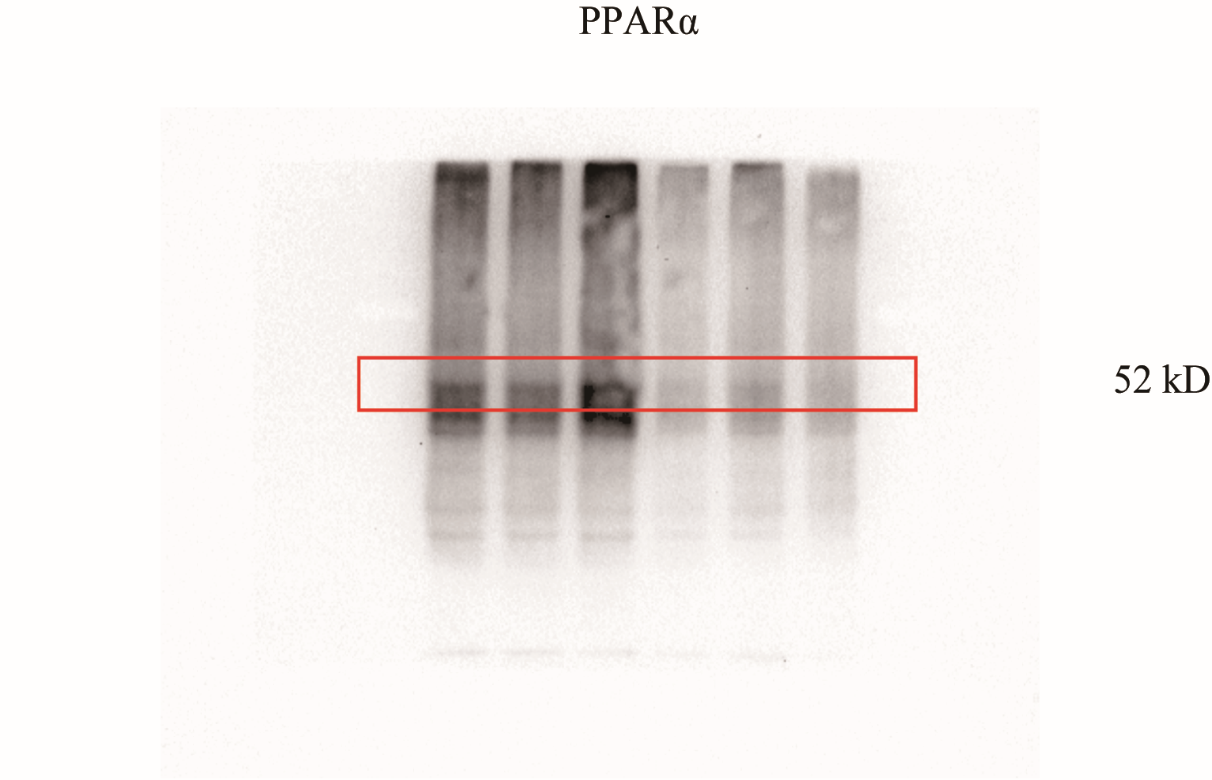


**Figure 6-PPARα**

**Figure 7A-Actin
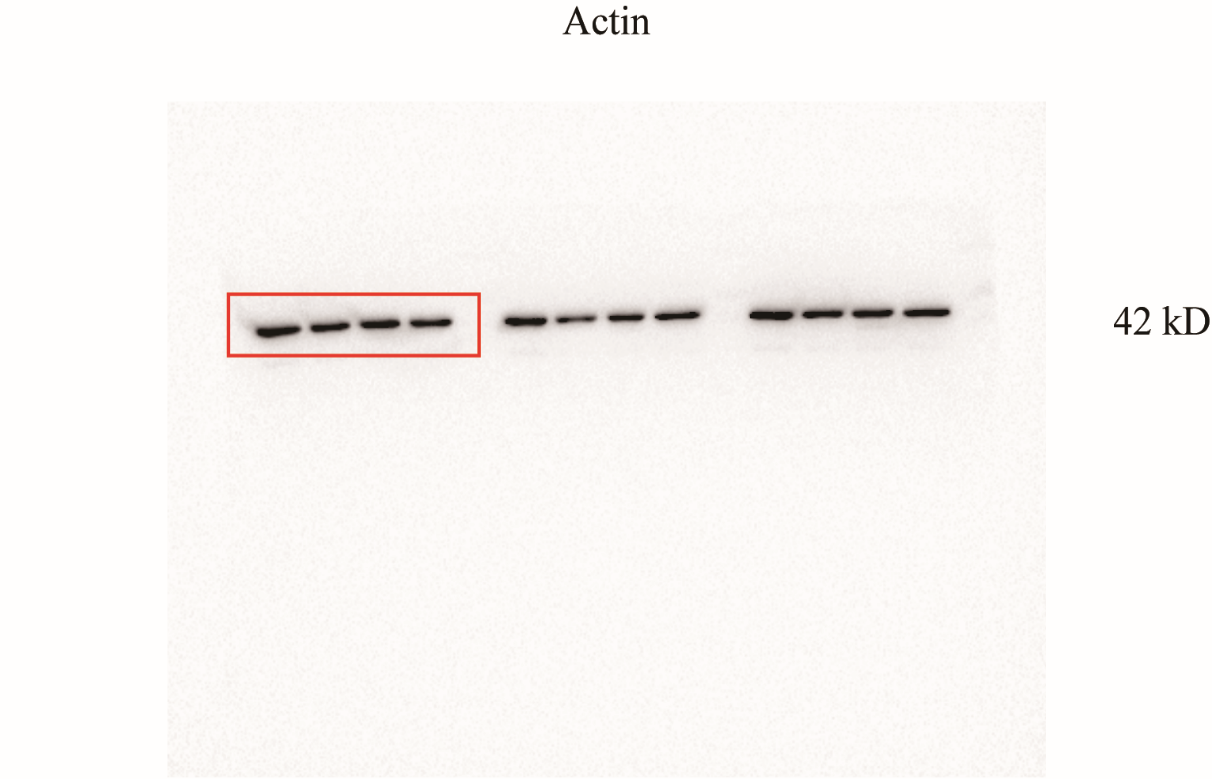
**

**Figure 7A-HtrA2/Omi
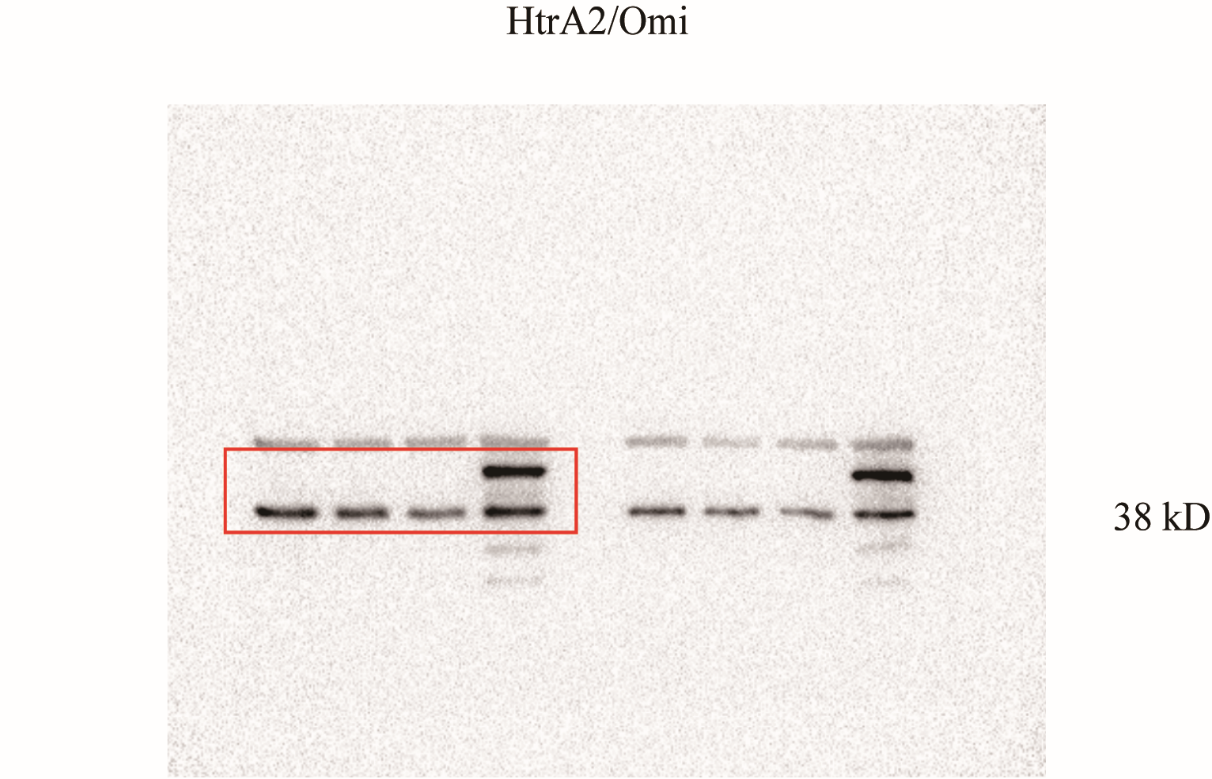
**

**Figure 7I-Actin
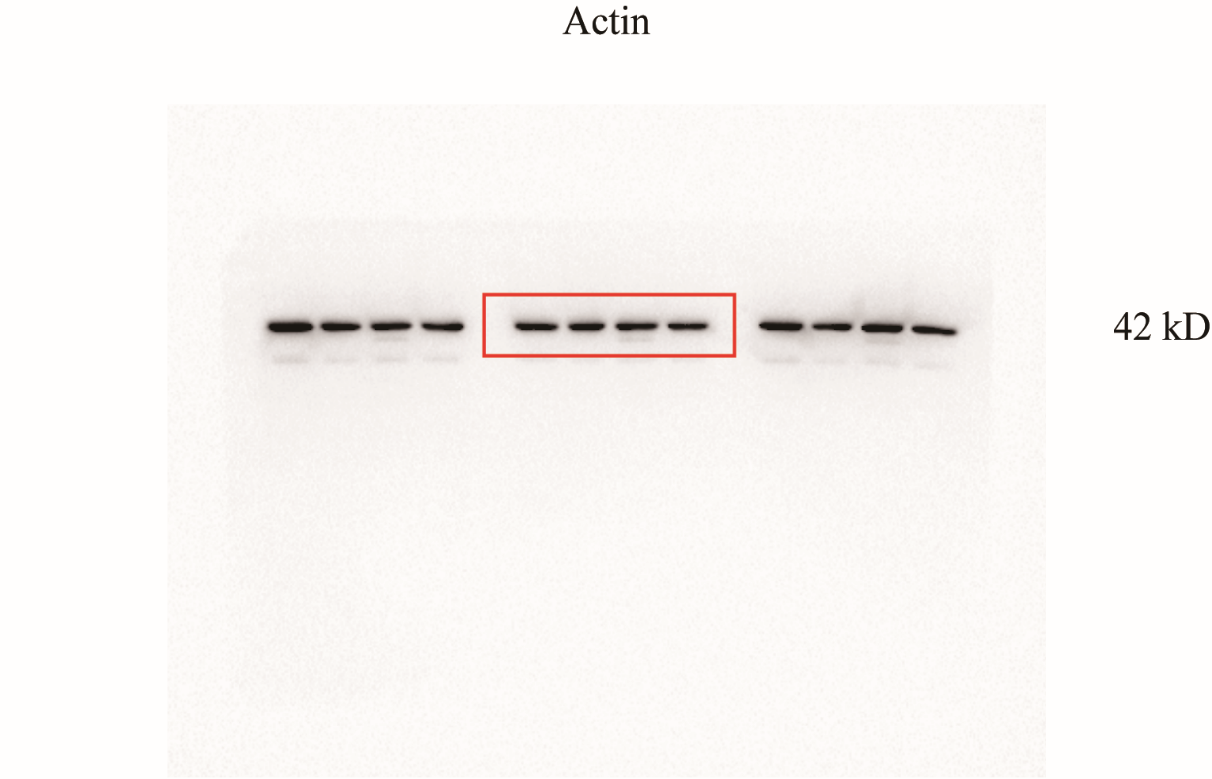
**

**Figure 7I-CPT1α
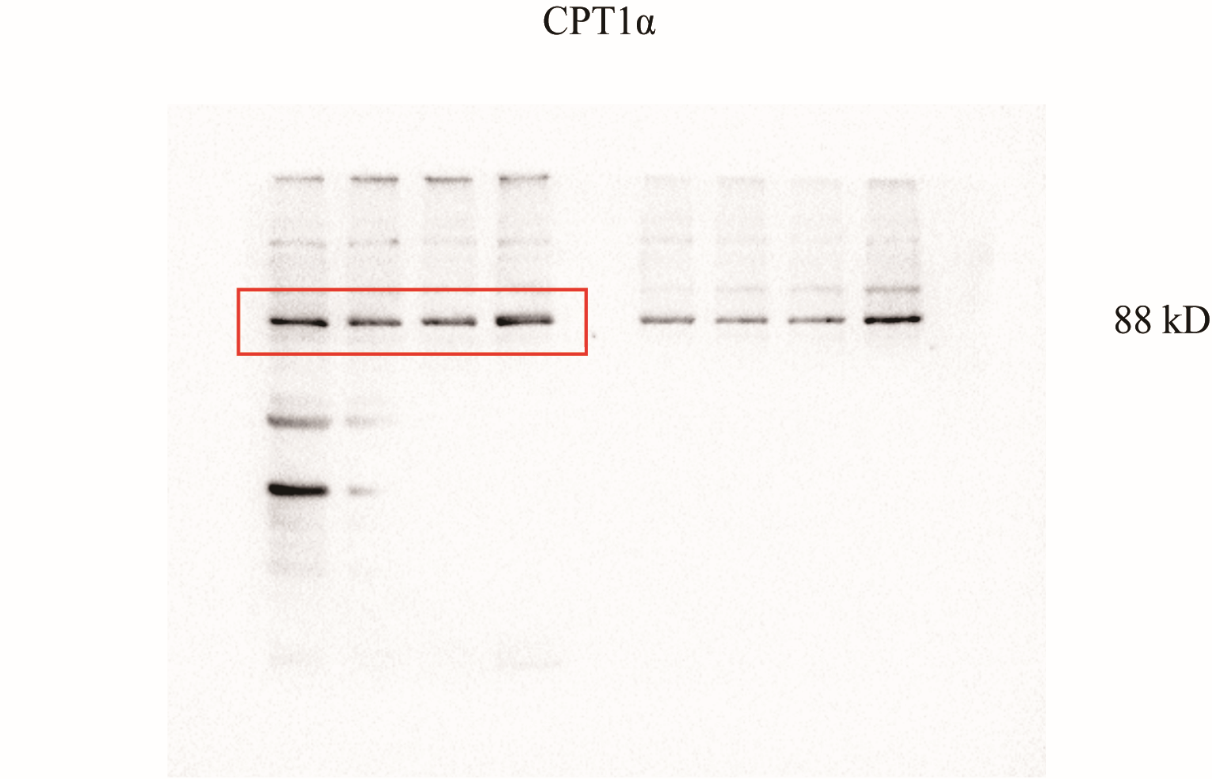
**

**Figure 7I-LC3I and LC3II
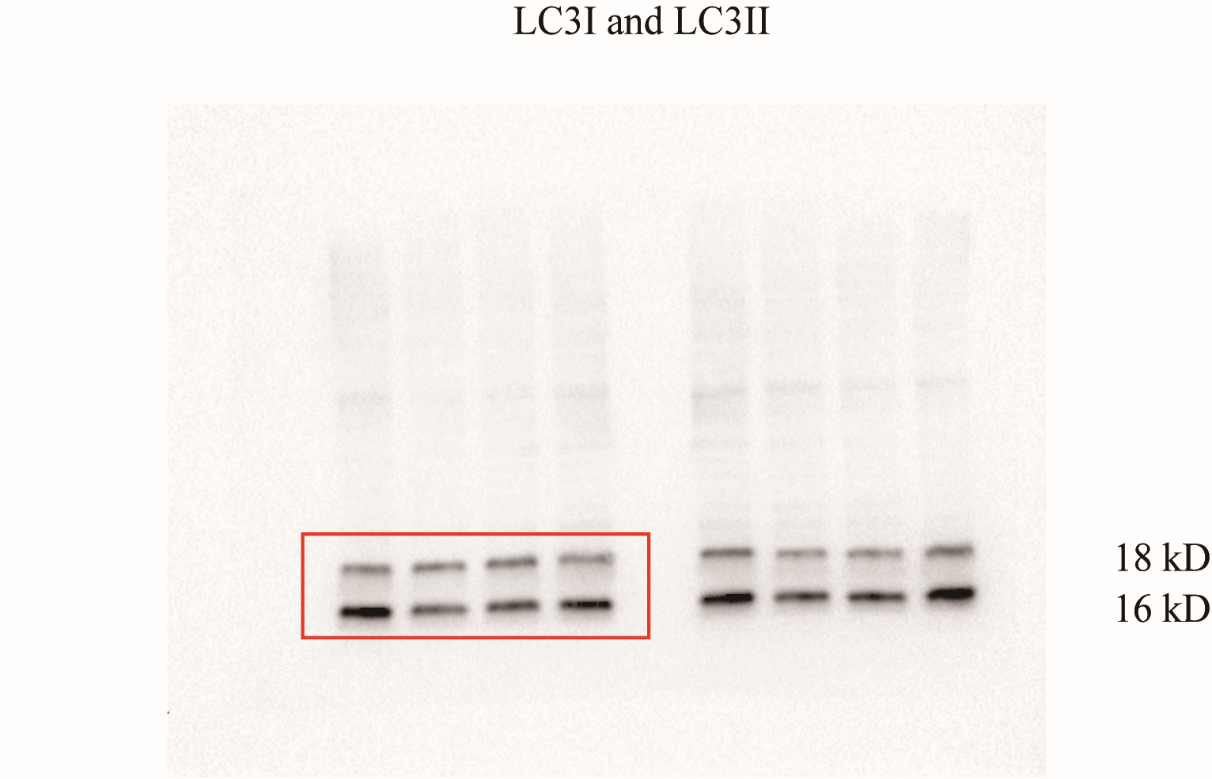
**

**Figure 7I-p62
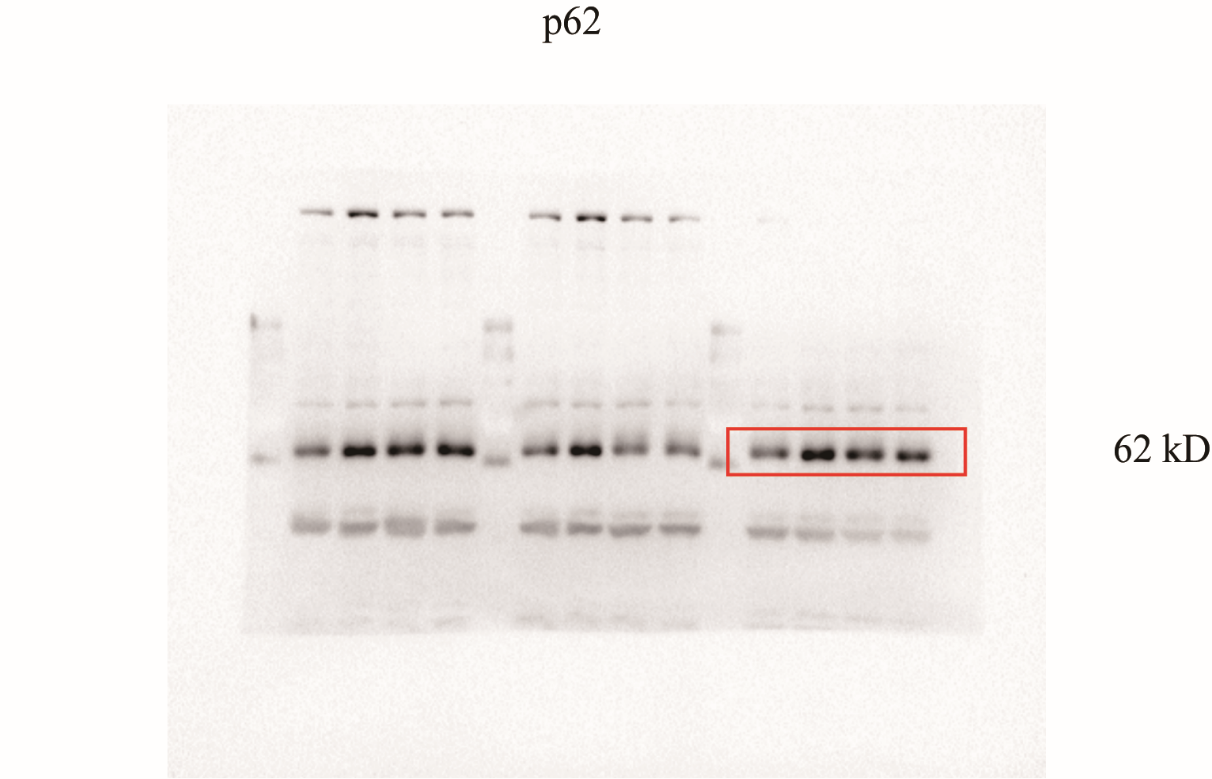
**

**Figure 7I-PPARα
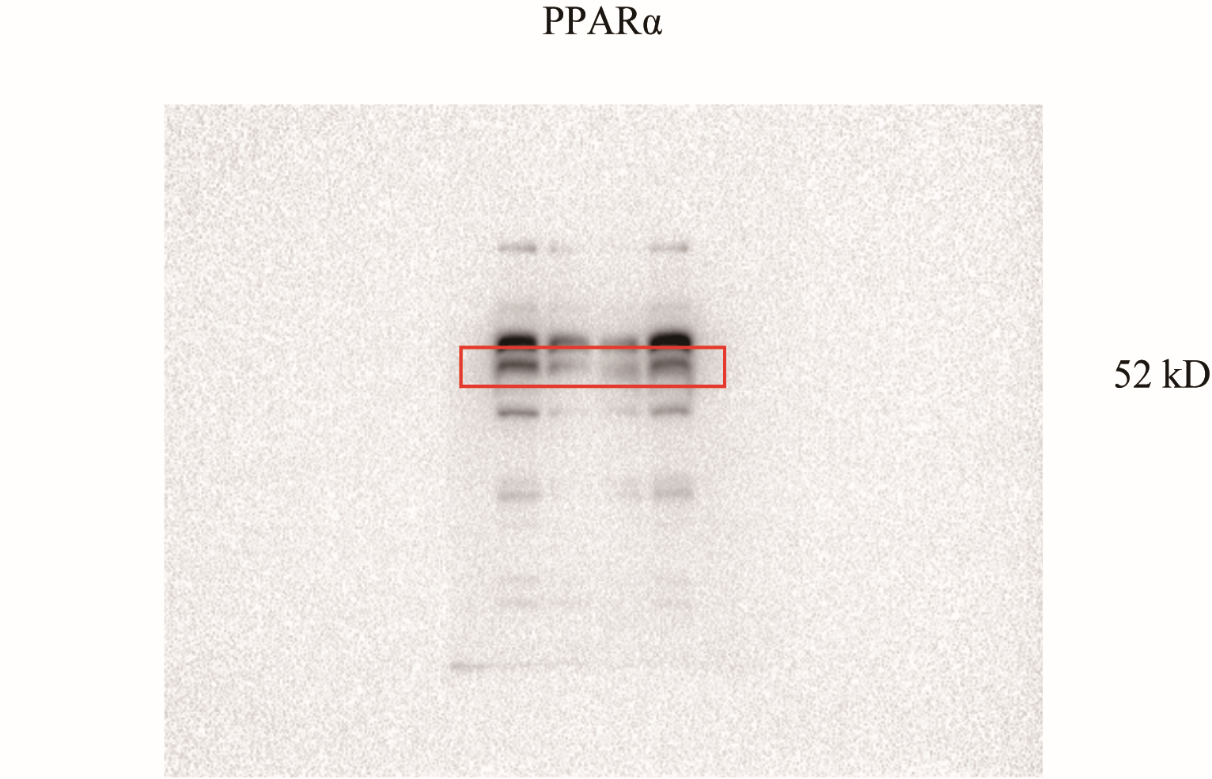
**
